# Dynein-2–driven intraciliary retrograde trafficking indirectly requires multiple interactions of IFT54 in the IFT-B complex with the dynein-2 complex

**DOI:** 10.1242/bio.059976

**Published:** 2023-06-28

**Authors:** Shunya Hiyamizu, Hantian Qiu, Yuta Tsurumi, Yuki Hamada, Yohei Katoh, Kazuhisa Nakayama

**Affiliations:** Department of Physiological Chemistry, Graduate School of Pharmaceutical Sciences, Kyoto University, Sakyo-ku, Kyoto 606-8501, Japan

**Keywords:** Cilia, Dynein-2, IFT-B complex, Intraflagellar transport

## Abstract

Within cilia, the dynein-2 complex needs to be transported as an anterograde cargo to achieve its role as a motor to drive retrograde trafficking of the intraflagellar transport (IFT) machinery containing IFT-A and IFT-B complexes. We previously showed that interactions of WDR60 and the DYNC2H1–DYNC2LI1 dimer of dynein-2 with multiple IFT-B subunits, including IFT54, are required for the trafficking of dynein-2 as an IFT cargo. However, specific deletion of the IFT54-binding site from WDR60 demonstrated only a minor effect on dynein-2 trafficking and function. We here show that the C-terminal coiled-coil region of IFT54, which participates in its interaction with the DYNC2H1–DYNC2LI1 dimer of dynein-2 and with IFT20 of the IFT-B complex, is essential for IFT-B function, and suggest that the IFT54 middle linker region between the N-terminal WDR60-binding region and the C-terminal coiled-coil is required for ciliary retrograde trafficking, probably by mediating the effective binding of IFT-B to the dynein-2 complex, and thereby ensuring dynein-2 loading onto the anterograde IFT trains. The results presented here agree with the notion predicted from the previous structural models that the dynein-2 loading onto the anterograde IFT train relies on intricate, multivalent interactions between the dynein-2 and IFT-B complexes.

## Teaser

**Summary:** Direct interaction of the dynein-2 complex with the IFT-B complex as an anterograde intraflagellar transport (IFT) cargo is indirectly required for dynein-2–driven retrograde IFT.

## INTRODUCTION

Primary cilia are microtubule-based, antenna-like appendages extending from the surface of most eukaryotic cells, and play crucial roles in receiving and integrating mechanical and chemical extracellular signals, such as the Hedgehog (Hh) family of morphogens (Anvarian et al., 2019; Kopinke et al., 2021). For these roles, specific proteins are present in the ciliary interior and on the ciliary membrane, including G protein-coupled receptors (GPCRs). The specific ciliary protein composition is maintained by the presence of the transition zone (TZ), which acts as a diffusion and permeability barrier at the ciliary base by restricting entry and exit of proteins (Garcia-Gonzalo and Reiter, 2017; Nachury and Mick, 2019). Defects in ciliary protein trafficking and abnormal ciliary protein localization therefore cause a broad range of genetic diseases, collectively referred to as the ciliopathies (Braun and Hildebrandt, 2017; Reiter and Leroux, 2017).

**Fig. 1.**
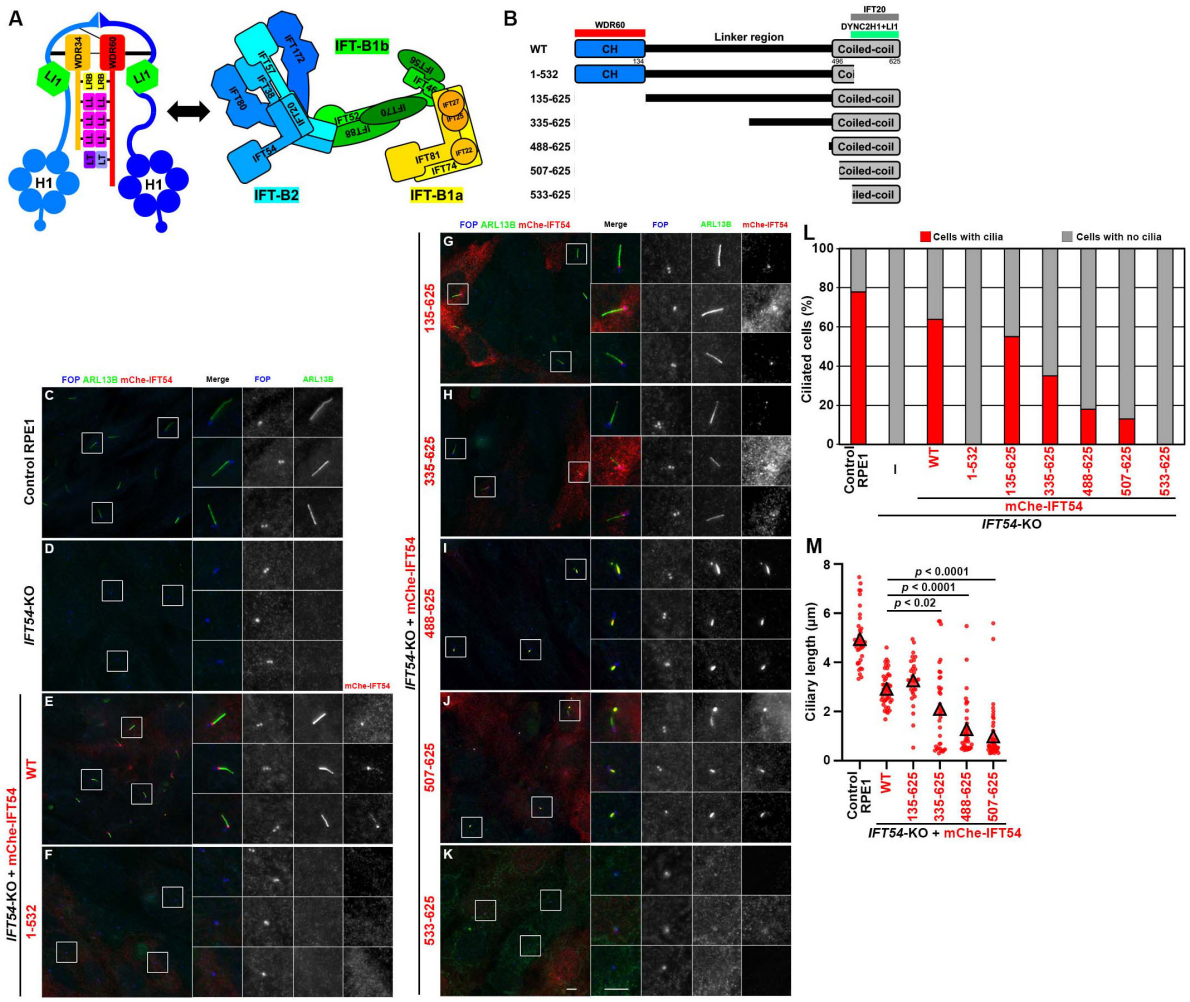
**The N-terminal CH domain and middle linker region of IFT54 are dispensable for ciliogenesis.** (A) Schematic representation of the architectures of the dynein-2 and IFT-B complexes. Individual IFT-B subunits are depicted with the first letter pointing to the N-terminal side. (B) Schematic representation of the structures of IFT54 constructs used in this study. Regions required for binding to WDR60, DYNC2H1–DYNC2LI1, and IFT20 determined in the previous study (Hiyamizu et al., 2023) are indicated. (C–K) Control RPE1 cells (C), *IFT54*-KO cells (D) and those stably expressing mCherry (mChe)-fused IFT54(WT) (E), IFT54(1–532) (F), IFT54(135–625) (G), IFT54(335–625) (H), IFT54(488–625) (I), IFT54(507–625) (J), or IFT54(533–625) (K) were cultured for 24 h under serum-starved conditions. The cells were immunostained for ARL13B, RFP, and FOP (recently renamed as CEP43). Enlarged (2.5-fold) images of the boxed regions are shown on the right side. Scale bars: 5 µm. (L) Cells shown in C–K (*n*=100) were classified into those with ARL13B-positive cilia (red) or with no cilia (gray), and the percentages were represented as stacked bar graphs. (M) Ciliary lengths of individual ciliated cells were measured and expressed as scatter plots (*n*=30). Triangles are mean ciliary lengths. Statistical significances were calculated using one-way ANOVA followed by Tukey's multiple comparison test.

The biogenesis, maintenance, and functions of cilia rely on intraflagellar transport (IFT), which was first found in *Chlamydomonas* flagella (Kozminski et al., 1995, 1993) but is highly conserved across eukaryotic species (Prevo et al., 2017). Not only is protein trafficking within cilia in the anterograde and retrograde directions, but the import and export of proteins across the TZ are mediated by IFT machinery, which includes the IFT-A and IFT-B complexes comprising six and 16 subunits, respectively (Nakayama and Katoh, 2020; Taschner and Lorentzen, 2016). The IFT-B complex is grouped into two subcomplexes; the IFT-B1 (IFT-B core) subcomplex composed of ten subunits (IFT22, IFT25, IFT27, IFT46, IFT52, IFT56, IFT70, IFT74, IFT81, and IFT88) and the IFT-B2 (IFT-B peripheral) subcomplex composed of six subunits (IFT20, IFT38, IFT54, IFT57, IFT80, and IFT172). These two subcomplexes are linked by two IFT-B1 and two IFT-B2 subunits (IFT52 and IFT88, and IFT38 and IFT57, respectively; see Fig. 1A, right) (Boldt et al., 2016; Katoh et al., 2016; Lacey et al., 2023; Petriman et al., 2022; Taschner et al., 2016).

Anterograde trafficking of the IFT machinery from the base to the tip of cilia is driven by heterotrimeric kinesin-II coupled to the IFT-B complex (Funabashi et al., 2018; Rosenbaum and Witman, 2002), whereas retrograde trafficking is believed to be driven by dynein-2/IFT dynein, probably coupled to the IFT-A complex (Nakayama and Katoh, 2020). The dynein-2 motor itself is also a multisubunit complex (Fig. 1A, left) (Vuolo et al., 2020; Webb et al., 2020). In the human dynein-2 structure clarified by cryoelectron microscopy (cryo-EM), two copies of the motor subunit DYNC2H1 associate with the light intermediate chain DYNC2LI1, which stabilizes DYNC2H1 (Taylor et al., 2015), and with the WD40 repeat domains of specific intermediate chains, namely WDR60 and WDR34 (recently renamed as DYNC2I1 and DYNC2I2, respectively) via the highly asymmetric N-terminal nonmotor region (Toropova et al., 2017, 2019; Webb et al., 2020). WDR60 and WDR34 are clumped by an array of the dimerized light chains (DYNLL1, DYNLL2, DYNLRB1, DYNLRB2, DYNLT1, DYNLT3, and DYNLT2B; the last of which was previously called TCTEX1D2) (Hamada et al., 2018; Toropova et al., 2019; Tsurumi et al., 2019). Intriguingly, variations in all of the dynein-2–specific subunits and those in all of the IFT-A subunits cause skeletal ciliopathies with a variety of clinical manifestations (McInerney-Leo et al., 2015; Reiter and Leroux, 2017; Schmidts, 2014; Zhang et al., 2018). In addition, variations of several IFT-B subunits, including IFT54 (also known as TRAF3IP1), IFT80, and IFT172, are also known to cause skeletal ciliopathies (Beales et al., 2007; Halbritter et al., 2013; Zhang et al., 2018). In this regard, our previous studies demonstrated the associations between defects in protein–protein interactions and ciliary defects caused by ciliopathy-associated variations of the IFT-A subunits IFT122 and IFT144, the dynein-2 subunits DYNC2LI1 and WDR34, and the IFT-B subunit IFT52 (Ishida et al., 2021, 2022; Qiu et al., 2022; Shak et al., 2023; Takahara et al., 2018).

To achieve its function as a retrograde motor, the dynein-2 complex must be transported to the ciliary tip as an anterograde IFT cargo. A model assembled by docking of the cryo-EM structure of the human dynein-2 complex (Toropova et al., 2019) onto the anterograde IFT train structure in *Chlamydomonas* flagella revealed by *in situ* cryoelectron tomography (cryo-ET) (Jordan et al., 2018), and an *in situ* cryo-ET study of assembling anterograde IFT trains at the *Chlamydomonas* flagellar base (van den Hoek et al., 2022) suggested that the dynein-2 complex has extensive contacts with the IFT-B complex of the anterograde train (Jordan and Pigino, 2021; Vuolo et al., 2020; Webb et al., 2020). In these structures, individual dynein-2 complexes are expected to span out multiple IFT-B unit repeats. Consistent with these structures, we have recently shown that multiple subunits of the dynein-2 complex interact predominantly with multiple subunits of the IFT-B2 subcomplex; individual dynein-2 subunits have contacts with distinct IFT-B2 subunits or even with different regions of the same IFT-B2 subunits (Hiyamizu et al., 2023). Our biochemical data are supported by a recently proposed model of the *Chlamydomonas* anterograde IFT train assembled using a combination of *in situ* cryo-ET data and the AlphaFold2 prediction, in which the dynein-2 complex contacts the IFT-B2 face of the IFT-B unit repeats (Lacey et al., 2023). Among the detected interactions between the dynein-2 and IFT-B complexes, the WDR60­–IFT54 interaction appeared to be a key interaction (Hiyamizu et al., 2023). However, a WDR60 construct with specific deletion of the IFT54-binding site showed apparently normal function similar to wild-type (WT) WDR60, although more extensive deletion substantially impaired the function of WDR60 as a subunit of the retrograde motor complex.

In the present study, we investigated the role of IFT54 in the dynein-2–IFT-B interaction, and indirectly in dynein-2­–driven retrograde trafficking. IFT54 has an N-terminal calponin-homology (CH) domain and a C-terminal coiled-coil region, which are linked via a middle, unstructured linker region. We previously showed that the CH domain and the coiled-coil region of IFT54 participate in its interactions with WDR60 and with the DYNC2H1–DYNC2LI1 dimer, respectively. The coiled-coil region of IFT54 is also essential for its interaction with IFT20 of the IFT-B complex (Hiyamizu et al., 2023). On the other hand, a previous study showed that *Chlamydomonas* IFT54 interacts with IFT dynein via its middle linker region, and that targeted disruption of this interaction impairs anterograde trafficking of IFT dynein (Zhu et al., 2021). Both in the AlphaFold model of the *Chlamydomonas* IFT-B complex validated using cross-linking/mass spectrometry analysis (Petriman et al., 2022), and in the model of the *Chlamydomonas* anterograde IFT train assembled using a combination of cryo-ET analysis and the AlphaFold predictions (Lacey et al., 2023), the long linker region of IFT54 was unmodeled, whereas the C-terminal region was found to form a parallel coiled-coil with IFT20, suggesting that the linker provides flexibility in the relative positioning of the N-terminal CH domain in the IFT-B complex. In this study, we therefore compared the phenotypes of *IFT54*-knockout (KO) cells expressing various deletion constructs of IFT54. We found that specific deletion of the IFT54 CH domain responsible for WDR60 binding had a marginal effect on dynein-2–driven retrograde trafficking. In clear contrast, the IFT54 C-terminal coiled-coil, which encompasses the region involved in binding to DYNC2H1–DYNC2LI1 and IFT20, was found to be essential for IFT-B function and for effective retrograde trafficking driven by the dynein-2 complex.

## RESULTS

### The N-terminal CH domain and middle linker region of IFT54 are dispensable for ciliogenesis

We and others recently identified interactions between the dynein-2 complex and the IFT-B complex (Hiyamizu et al., 2023; Vuolo et al., 2018). In agreement with the current model of the dynein-2 complex docked with the anterograde IFT train (Jordan et al., 2018; Lacey et al., 2023; Toropova et al., 2019), these multiple interactions were identified between dynein-2 and the IFT-B2 subunits. In particular, WDR60 and the DYNC2H1–DYNC2LI1 dimer from dynein-2, and the IFT-B2 subunits IFT54 and IFT57 make substantial contributions to the dynein-2–IFT-B interaction (Hiyamizu et al., 2023); a *Chlamydomonas* study also supported the important role of IFT54 in binding to dynein-2 during anterograde IFT (Zhu et al., 2021). Both IFT54 and IFT57 have N-terminal CH domains and C-terminal coiled-coil regions, which are connected via relatively long linker regions. The coiled-coil regions of IFT54 and IFT57 form parallel coiled-coils with IFT20 and IFT38, respectively (Katoh et al., 2016; Taschner et al., 2016; Zhu et al., 2017); the IFT20–IFT54 and IFT38–IFT57 dimers then form an antiparallel tetramer (schematically shown in Fig. 1A, right) (Lacey et al., 2023; Petriman et al., 2022). We showed that IFT54 interacts with WDR60 and DYNC2H1–DYNC2LI1 via its CH domain and the coiled-coil region, respectively (see Fig. 1B and Table 1); as described above, the IFT54 coiled-coil region is also crucial for parallel coiled-coil formation with IFT20 (Hiyamizu et al., 2023; Omori et al., 2008; Petriman et al., 2022). On the other hand, the middle linker region of IFT54 is predicted to be unstructured by AlphaFold predictions (Lacey et al., 2023; Petriman et al., 2022), and is likely to flexibly interact with the dynein-2 subunits.

**Table 1.**
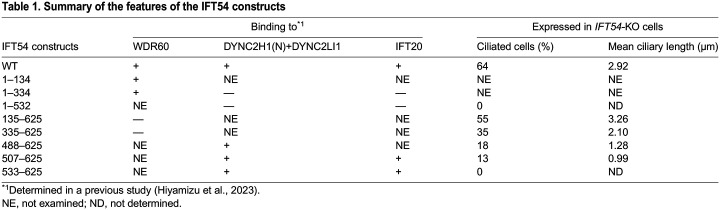
Summary of the features of the IFT54 constructs

We here investigated the roles of the interactions of IFT54 with the dynein-2 subunits and IFT20. To this end, we first established *IFT54*-KO cells from human telomerase reverse transcriptase-immortalized retinal pigment epithelial 1 (hTERT-RPE1) cells (Fig. S1). In good agreement with a previous study on a *Chlamydomonas ift54*-null mutant (Zhu et al., 2017), *IFT54*-KO cells demonstrated a cilia-lacking phenotype depicted by staining for ARL13B (Fig. 1C,D). We then expressed various IFT54 constructs in *IFT54*-KO cells. The stable expression of mCherry-IFT54(WT) restored ciliogenesis (Fig. 1E; also see Fig. 1L), excluding the possibility that the cilia-lacking phenotype of *IFT54*-KO cells was a result of an off-target effect, although we could not determine the exact disruption site in one of the *IFT54* alleles (see the legend for Fig. S1B). Comparable to IFT54(WT), ciliogenesis was also restored by the stable expression of mCherry-fused IFT54(135–625) (Fig. 1G; also see Fig. 1L,M), which lacks the CH domain responsible for WDR60 binding (see Fig. 1B and Table 1); this observation is in line with a previous study using the *Chlamydomonas ift54* mutant lacking the CH domain (Zhu et al., 2017). Thus, the IFT54 CH domain is dispensable for ciliogenesis, although a previous study suggested that the CH domain of *Chlamydomonas* IFT54 can bind the αβ-tubulin dimer *in vitro* (Taschner et al., 2016).

**Fig. 2.**
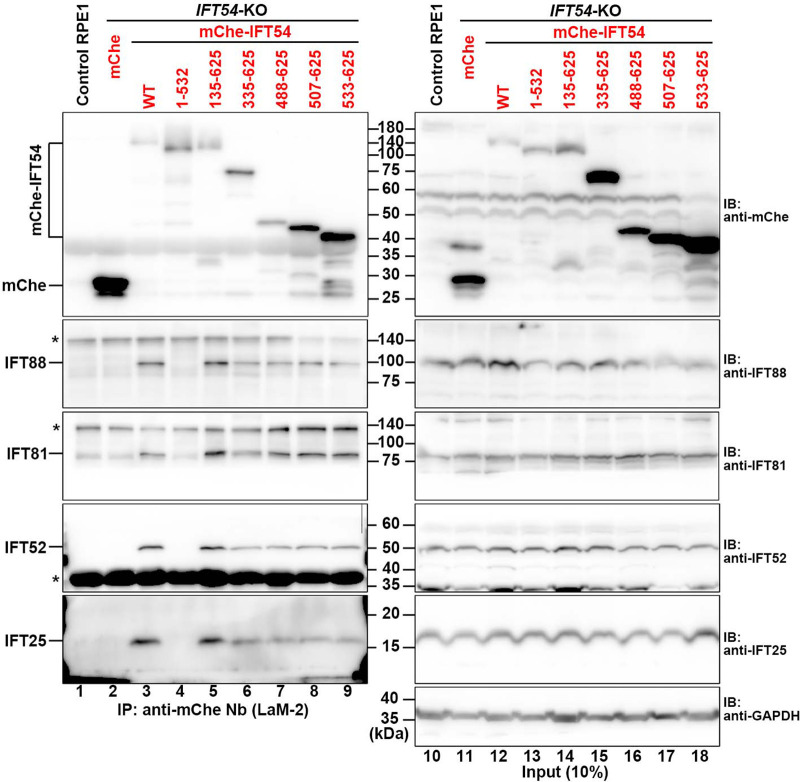
**Incorporation of mCherry-IFT54 constructs into the IFT-B complex.** Endogenous IFT-B subunits were subjected to coimmunoprecipitation with mCherry (mChe)-IFT54 constructs. Lysates prepared from control RPE1 cells, or *IFT54*-KO cells stably expressing mChe or mChe-fused IFT54 constructs as indicated were processed for immunoprecipitation with GST-tagged anti-mChe Nb (LaM-2 version) prebound to glutathione-Sepharose beads. The precipitates were subjected to SDS-PAGE and immunoblotting analysis using antibodies against mChe, IFT88, IFT81, IFT52, IFT25, and GAPDH. Asterisks indicate the positions of nonspecific bands.

In clear contrast to IFT54(WT) and IFT54(135–625), the stable expression of mCherry-fused IFT54(1–532), which lacks a part of the coiled-coil region responsible for binding to IFT20 and the DYNC2H1–DYNC2LI1 dimer (see Fig. 1B and Table 1), could not rescue the ciliogenesis defect of *IFT54*-KO cells (Fig. 1F; also see Fig. 1L). This was probably because IFT54(1–532) is no longer incorporated into the IFT-B complex owing to its lack of binding to IFT20, for two reasons. Firstly, mCherry-IFT54(1–532) signals were not detected around the basal body marked by FOP (recently renamed as CEP43) (Fig. 1F). Secondly, using the anti-mCherry nanobody (Nb), mCherry-fused IFT54(WT) and IFT54(135–625), but not IFT54(1–532), expressed in *IFT54*-KO cells coimmunoprecipitated the endogenous IFT-B1 subunits IFT88, IFT81, IFT52, and IFT25 (Fig. 2, lanes 3–5), none of which has direct contact with IFT54 in the IFT-B complex (see Fig. 1A, right) (Lacey et al., 2023; Petriman et al., 2022). Note that the expression levels of the IFT54 constructs containing the middle linker region, namely IFT54(WT), IFT54(1–532), and IFT54(135–625), were relatively low (Fig. 2, top panel, lanes 12–14) compared with the other constructs (lanes 15–18), probably because the unstructured linker region destabilizes the IFT54 protein in the cell lysates. Despite this relative instability, mCherry-fused IFT54(WT) and IFT54(135–625) were detected around the ciliary base (Fig. 1E, G, rightmost panels), and coprecipitated endogenous IFT-B1 proteins using anti-mCherry Nb (Fig. 2, lanes 3 and 5).

We also observed cilia formation in *IFT54*-KO cells expressing IFT54 constructs that were further truncated from the N-terminus [IFT54(335–625), IFT54(488–625), and IFT54(507–625)]. With longer truncations of the linker region from the N-terminus, the ciliogenesis efficiency of *IFT54*-KO cells expressing the IFT54 construct decreased gradually (Fig. 1L), and the ciliary length tended to be shorter (Fig. 1H–J; also see Fig. 1M). In *IFT54*-KO cells expressing mCherry-fused IFT54(533–625), which retains the binding abilities to IFT20 and DYNC2H1–DYNC2LI1, virtually no cilia were formed (Fig. 1K; also see Fig. 1L). As mCherry-IFT54(533–625) could coimmunoprecipitate endogenous IFT-B1 proteins (Fig. 2, lane 9), the IFT54(533–625) construct is likely to be incorporated into the IFT-B complex. The reason for the cilia-lacking phenotype of IFT54(533–625)-expressing *IFT54*-KO cells will be discussed later (see Discussion).

### IFT54-KO cells expressing N-terminally truncated IFT54 constructs are impaired in retrograde trafficking of the IFT machinery

**Fig. 3.**
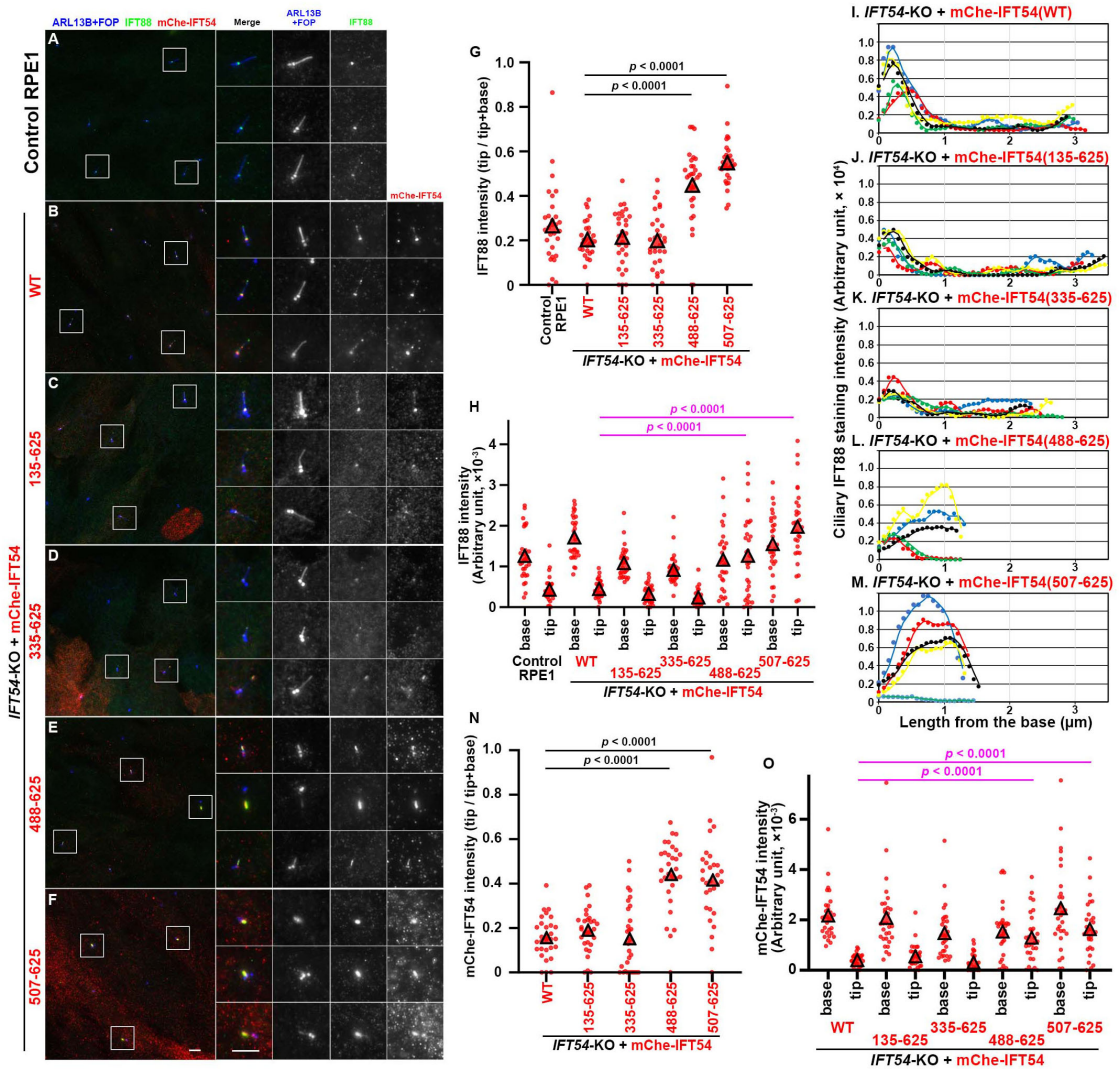
**IFT88 accumulation in the distal tip region of cilia in *IFT54*-KO cells expressing truncated IFT54.** (A–F) Control RPE1 cells (A), *IFT54*-KO cells stably expressing mCherry (mChe)-fused IFT54(WT) (B), IFT54(135–625) (C), IFT54(335–625) (D), IFT54(488–625) (E), or IFT54(507–625) (F) were serum-starved for 24 h and immunostained for IFT88, RFP, and ARL13B+FOP. Enlarged (2.5-fold) images of the boxed regions are shown on the right side. Scale bars: 5 µm. (G,H) The IFT88 staining intensities in the ciliary base and tip regions of individual ciliated cells were measured (*n*=30) and expressed as scatter plots (H). In G, relative IFT88 staining intensities in the ciliary base and tip regions were estimated, and the ratio of tip/(tip+base) was represented as scatter plots. Triangles indicate the means of the experiments. Statistical significances were calculated using one-way ANOVA followed by Tukey's multiple comparison test. (I–M) Line scans of IFT88 staining intensities along individual cilia of *IFT54*-KO cells stably expressing mChe-fused IFT54(WT) (I), IFT54(135–625) (J), IFT54(335–625) (K), IFT54(488–625) (L), or IFT54(507–625) (M). Line scans of cilia with lengths that fall within 10% of the mean length are shown (*n*=5). (N,O) Staining intensities of the IFT54 constructs in the ciliary base and tip regions of individual ciliated cells were measured (*n*=30) and represented as scatter plots as in G and H.

We then investigated the localization of the components of the IFT machinery in *IFT54*-KO cells expressing the various mCherry-IFT54 constructs; note that in these experiments, we omitted cells that did not form cilia [*IFT54*-KO cells expressing mCherry-IFT54(1–532) and those expressing mCherry-IFT54(533–625)]. In control RPE1 cells and *IFT54*-KO cells expressing mCherry-IFT54(WT), the IFT-B subunit IFT88 was mainly detected at the ciliary base, and a minor fraction was detected at the tip (Fig. 3A,B; also see Fig. 3G,H,I). In *IFT54*-KO cells expressing mCherry-fused IFT54(135–625) or IFT54(335–625) (Fig. 3C,D), the ratio of tip-to-(base+tip) for IFT88 signals was not significantly different from that in control RPE1 cells and in *IFT54*-KO cells expressing mCherry-IFT54(WT) (Fig. 3G,H). By contrast, in *IFT54*-KO cells expressing mCherry-fused IFT54(488–625) or IFT54(507–625), IFT88 was significantly enriched around the tip (in the distal region) of shortened cilia (Fig. 3E,F; also see Fig. 3G,H). Line scanning of IFT88 staining images along cilia confirmed that IFT88 was considerably enriched within cilia, in particular, in the distal region of severely shortened cilia of *IFT54*-KO cells expressing mCherry-fused IFT54(488–625) or IFT54(507–625) (Fig. 3L,M). The localization of the expressed IFT54 constructs at the ciliary base and tip almost completely overlapped with that of endogenous IFT88. Namely, mCherry-fused IFT54(WT), IFT54(135–625), and IFT54(335–625) were mainly found at the base, and a minor fraction at the tip (Fig. 3B–D), whereas mCherry-fused IFT54(488–625) and IFT54(507–625) accumulated in the distal region of shortened cilia (Fig. 3E,F, also see Fig. 3N,O).

**Fig. 4.**
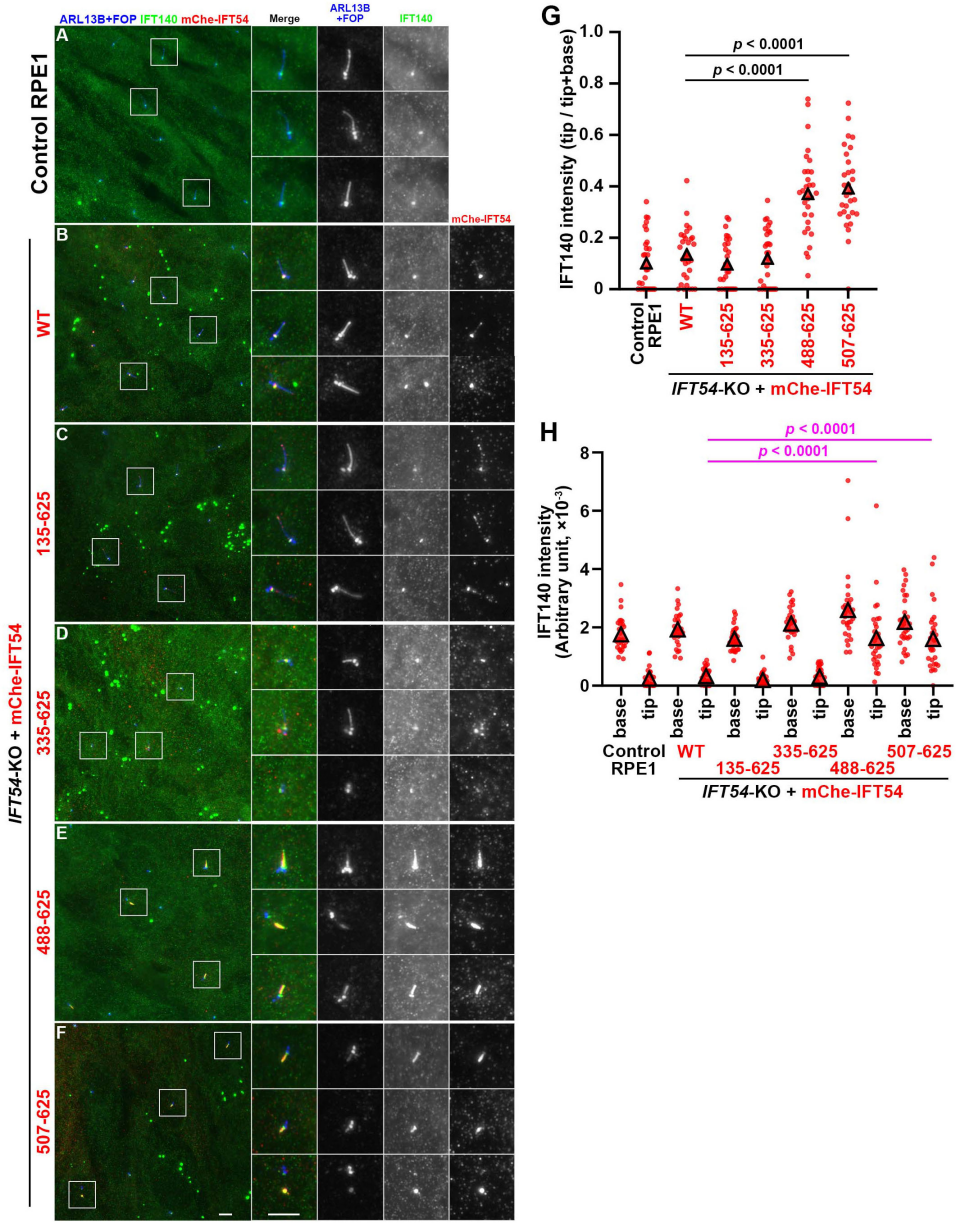
**IFT140 accumulation in the distal tip region of cilia in *IFT54*-KO cells expressing truncated IFT54.** (A–F) Control RPE1 cells (A), *IFT54*-KO cells stably expressing mCherry (mChe)-fused IFT54(WT) (B), IFT54(135–625) (C), IFT54(335–625) (D), IFT54(488–625) (E), or IFT54(507–625) (F) were serum-deprived for 24 h and immunostained for IFT140, RFP, and ARL13B+FOP. Boxed regions were 2.5-fold enlarged and shown on the right side. Scale bars: 5 µm. (G,H) The IFT140 staining intensities in the ciliary base and tip regions of individual ciliated cells were measured (*n*=30) and expressed as scatter plots (H). In G, relative IFT140 staining intensities in the ciliary base and tip regions were estimated, and the ratio of tip/(tip+base) was expressed as scatter plots. Triangles indicate the means of the experiments. Statistical significances were calculated using one-way ANOVA followed by Tukey's multiple comparison test.

Localization of the IFT-A subunit IFT140 also substantially overlapped with that of the mCherry-fused IFT54 constructs. In *IFT54*-KO cells expressing mCherry-fused IFT54(WT), IFT54(135–625), and IFT54(335–625), IFT140 was mainly localized at the ciliary base (Fig. 4B–D) as in control RPE1 cells (Fig. 4A); note that the anti-IFT140 antibody that was used often stains nonspecific cytoplasmic and nuclear dots, as described previously (Hirano et al., 2017; Shak et al., 2023; Takahara et al., 2018). By contrast, *IFT54*-KO cells expressing mCherry-fused IFT54(488–625) or IFT54(507–625) demonstrated considerable enrichment of IFT140 in the distal region of severely shortened cilia, like as the mCherry-fused IFT54 constructs themselves (Fig. 4E,F, also see Fig. 4G,H). The accumulation of IFT-B and IFT-A components in the distal region of short cilia resembles that observed in *DNYC2LI1*-KO cells (Hiyamizu et al., 2023; Qiu et al., 2022) and in *DYNC2H1*-KO cells (Wu et al., 2017), but not that in *WDR60*-KO and *WDR34*-KO cells (Hamada et al., 2018; Hiyamizu et al., 2023; Tsurumi et al., 2019; Vuolo et al., 2018; Wu et al., 2017). It is therefore likely that IFT54(488–625)-expressing and IFT54(507–625)-expressing *IFT54*-KO cells demonstrated very short cilia, and considerable accumulation of IFT-B and IFT-A within cilia due to the severely impaired function of dynein-2 as a retrograde motor (see Discussion).

### IFT54-KO cells expressing N-terminally truncated IFT54 constructs are defective in the export of GPCRs from cilia

**Fig. 5.**
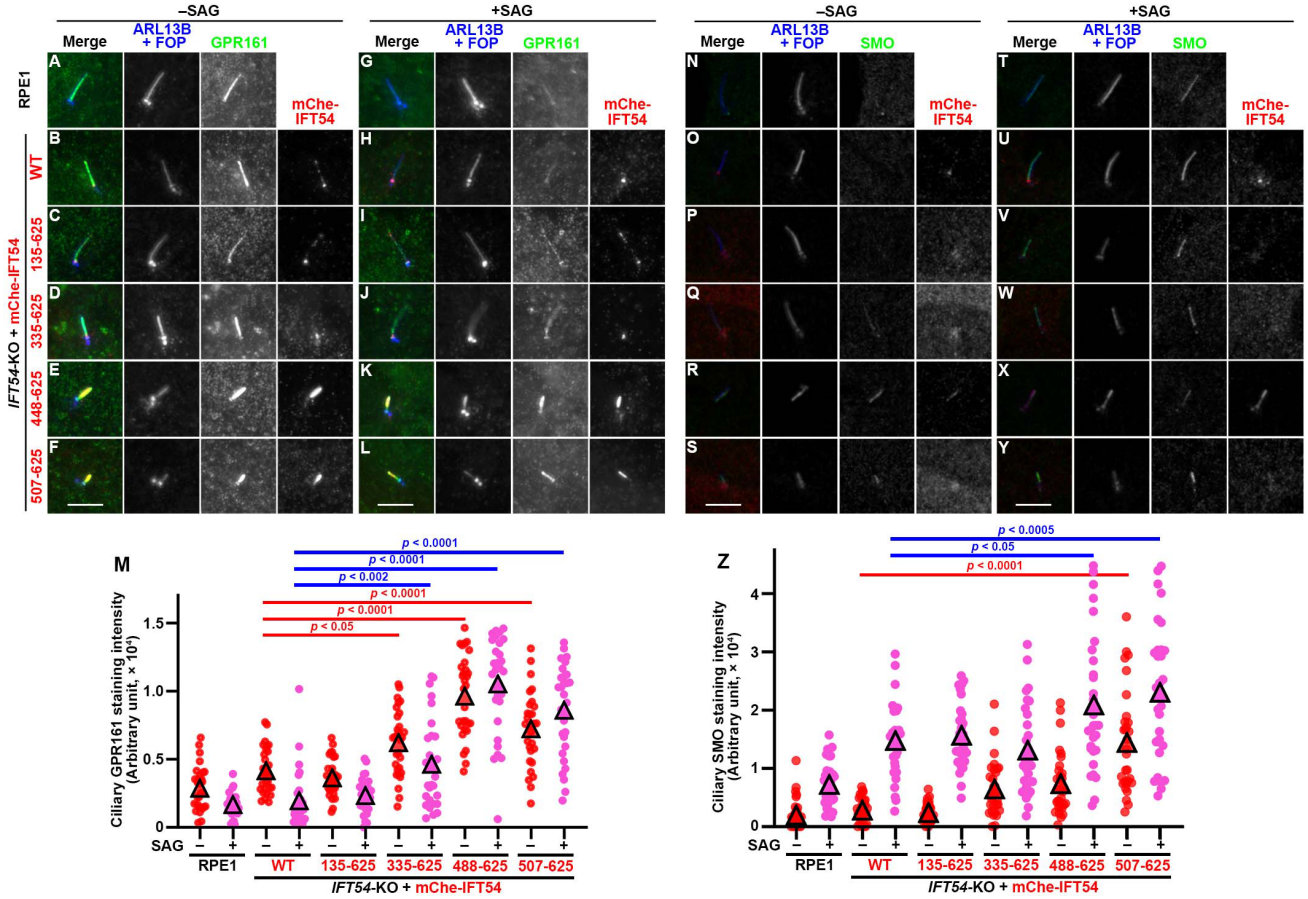
**Defects in export of GPCRs from cilia in *IFT54*-KO cells expressing truncated IFT54.** (A–L, N–Y) Control RPE1 cells (A,G,N,T), and *IFT54*-KO cells stably expressing mCherry (mChe)-fused IFT54(WT) (B,H,O,U), IFT54(135–625) (C,I,P,V), IFT54(335–625) (D,J,Q,W), IFT54(488–625) (E,K,R,X), or IFT54(507–625) (F,L,S,Y) were serum-deprived for 24 h and then incubated for a further 24 h in the absence (–SAG; A–F, N–S) or presence (+SAG; G–L, T–Y) of 200 nM SAG. The cells were immunostained for either GPR161 (A–L) or SMO (N–Y), and RFP and ARL13B+FOP. Scale bars: 5 µm. (M,Z) The ciliary staining intensities of GPR161 (M) and SMO (Z) in individual ciliated cells were measured and expressed as scatter plots (*n*=30). Triangles indicate the mean staining intensities. Statistical significances were calculated using one-way ANOVA followed by Tukey's multiple comparison test.

We then investigated changes in the localization of GPR161, which is a class A GPCR that negatively regulates Hh signaling (Kopinke et al., 2021) in response to stimulation of the Hh pathway, as the retrograde trafficking and/or export from cilia of GPR161 mediated by the BBSome coupled with the IFT machinery is thought to be powered by dynein-2. In control RPE1 cells, GPR161 was found on the ciliary membrane under basal conditions, whereas it was exported from cilia when the Hh pathway was stimulated by the treatment of cells with Smoothened agonist (SAG) (Fig. 5A,G). Essentially the same results were obtained for *IFT54*-KO cells expressing mCherry-IFT54(WT) (Fig. 5B,H), and those expressing mCherry-IFT54(135–625) (Fig. 5C,I). By contrast, in *IFT54*-KO cells expressing mCherry-IFT54(335–625), ciliary GPR161 levels were slightly, but significantly, high in both the basal and SAG-stimulated states compared with the levels in control RPE1 cells and in IFT54(WT)-expressing *IFT54*-KO cells (Fig. 5D,J, also see Fig. 5M). The abnormal enrichment of GPR161 within cilia under both basal and SAG-stimulated conditions was further enhanced in *IFT54*-KO cells expressing either mCherry-fused IFT54(488–625) (Fig. 5E,K) or IFT54(507–625) (Fig. 5F,L, also see Fig. 5M).

We also analyzed SAG-induced changes in the localization of Smoothened (SMO), which is a class F GPCR that positively regulates Hh signaling (Kopinke et al., 2021). In control RPE1 cells and in *IFT54*-KO cells expressing mCherry-IFT54(WT), SMO was not found within cilia under basal conditions (Fig. 5N,O) due to constitutive transport both in and out of cilia in the steady state (Kopinke et al., 2021). However, SMO was enriched within cilia in response to SAG treatment (Fig. 5T,U, also see Fig. 5Z). Similar results of changes in SMO localization were obtained for *IFT54*-KO cells expressing mCherry-IFT54(135–625) (Fig. 5P,V,Z). In *IFT54*-KO cells expressing mCherry-IFT54(335–625), the basal ciliary level of SMO tended to be slightly high, although not statistically significant (Fig. 5Q,Z). In *IFT54*-KO cells expressing either mCherry-fused IFT54(488–625) (Fig. 5R,X) or IFT54(507–625) (Fig. 5S,Y, also see Fig. 5Z), the basal and SAG-stimulated levels of ciliary SMO were higher than in cells expressing IFT54(WT). Thus, defects in the export of GPR161 and SMO from cilia appeared to correlate with the impairment in dynein-2–driven retrograde trafficking.

## DISCUSSION

The structural model of the *Chlamydomonas* anterograde IFT train suggests that the heavy chains of the dynein-2 complex contact the IFT-B2 face of the polymerized IFT-B complexes (Lacey et al., 2023). However, due to the resolution limit and the presence of unmodeled regions in the cryo-ET structure, details of the dynein-2–IFT-B2 interactions have not been fully elucidated. On the other hand, we biochemically demonstrated that multiple dynein-2 subunits and multiple IFT-B2 subunits participate in the interactions between the dynein-2 and IFT-B complexes (Hiyamizu et al., 2023). In particular, we found that IFT54 of the IFT-B2 subcomplex can interact with WDR60 and the DYNC2H1–DYNC2LI1 dimer via its N-terminal CH domain and the C-terminal coiled-coil region, respectively (schematically shown in Fig. 1B) (Hiyamizu et al., 2023), and a *Chlamydomonas* study showed that IFT54 plays an important role in the binding of IFT-B to IFT dynein (Zhu et al., 2021). In this context, it is of note that, probably due to the presence of the flexible linker region, the IFT54 CH domain was unmodeled in the cryo-ET structure of the anterograde train (Lacey et al., 2023), and in the structural model of the IFT-B complex using AlphaFold prediction with validation by chemical cross-linking/mass-spectrometry (Petriman et al., 2022). Therefore, we here analyzed the roles of the IFT54 interactions with dynein-2 subunits by observing the phenotypes of *IFT54*-KO cells expressing various IFT54 constructs.

*IFT54*-KO cells expressing IFT54(135–625), which specifically lacks the CH domain that is responsible for its binding to WDR60 (Fig. 1B and Table 1) (Hiyamizu et al., 2023) and was reported to be able to bind tubulin *in vitro* (Taschner et al., 2016), appeared normal compared with cells expressing IFT54(WT), with respect to the various criteria analyzed. Given that dynein-2 subunits have multiple contacts with IFT-B2 subunits (Hiyamizu et al., 2023; Lacey et al., 2023) and that a WDR60 construct specifically lacking just the IFT54-binding site rescued ciliary defects of *WDR60*-KO cells, similar to WDR60(WT) (Hiyamizu et al., 2023), it is likely that the lack of binding of IFT54 to WDR60 alone does not substantially affect trafficking of the dynein-2 complex as an anterograde IFT cargo. *IFT54*-KO cells expressing IFT54(335–625) also appeared almost normal, although ciliogenesis efficiency and ciliary length were significantly, although slightly, decreased compared with cells expressing IFT54(WT) or IFT54(135–625). In clear contrast, *IFT54*-KO cells expressing a construct lacking almost the entire linker region as well as the CH domain, i.e. IFT54(488–625) or IFT54(507–625), demonstrated considerable accumulation of IFT-B and IFT-A components in the distal region of severely shortened cilia. In IFT54(488–625)-expressing and IFT54(507–625)-expressing *IFT54*-KO cells, dynein-2 is likely to be severely impaired with respect to driving retrograde trafficking due to its impaired trafficking as an anterograde cargo, although the dynein-2 complex itself should be intact, in view of the fact that cells lacking the motor subunit DYNC2H1 and those lacking DYNC2LI1, which stabilizes DYNC2H1 (Taylor et al., 2015), demonstrate similar defects in retrograde trafficking (Hiyamizu et al., 2023; Qiu et al., 2022; Wu et al., 2017). On the other hand, the IFT54(533–625) construct could not restore the ciliogenesis of *IFT54*-KO cells (Fig. 1K,L), even though it retained the ability to be incorporated into the IFT-B complex via interacting with IFT20 (Fig. 2, lane 9). Although we do not know the exact reason for these results of IFT54(533–625), the deleted region may have a role in the IFT-B complex other than incorporation of IFT54 into the complex, such as lateral interactions of the IFT-B repeats, as the region was unmodeled in structural studies (Lacey et al., 2023; Petriman et al., 2022).

The docking model of the human dynein-2 complex to the *Chlamydomonas* anterograde IFT train (Jordan et al., 2018; Toropova et al., 2019) and the cryo-ET structure of the *Chlamydomonas* anterograde train clarified with the aid of AlphaFold prediction (Lacey et al., 2023) predicted that a single dynein-2 complex has multiple contacts with the IFT-B2 side of the polymerized IFT-B complexes when transported as an anterograde cargo. On the other hand, we showed that multiple dynein-2 subunits (WDR60, the DYNC2H1–DYNC2LI2 dimer, and WDR34) can interact with multiple IFT-B2 subunits (IFT54, IFT57, IFT172, IFT80, and IFT38) (Hiyamizu et al., 2023). Thus, the loading of dynein-2 onto the anterograde IFT train appears to rely on intricate, multivalent interactions between the dynein-2 subunits and the IFT-B subunits, and disruption of a single interaction, such as that between WDR60 and IFT54, would have a marginal effect on dynein-2 loading as a whole. However, ciliary retrograde trafficking is likely to be impaired if considerable changes occur in the IFT-B complex that render the intact dynein-2 complex unable to be loaded onto the anterograde train. To corroborate the above reasoning, we attempted to track the movement of mCherry-fused IFT54 constructs expressed in *IFT54*-KO cells within the cilia by total internal reflection fluorescence microscopy but have been unsuccessful to date due to the weak intensity of the fluorescence signals. In the future, the use of a fluorescent protein tag, such as mNeonGreen, which has strong fluorescence signals, or StayGold, which is less likely to be photobleached (Hirano et al., 2022), may enable us to observe the movement of the IFT54 mutants.

It is interesting to note a case study that reported five unrelated families (eight patients) of nephronophthisis (NPHP)-related ciliopathies with IFT54 variations (Bizet et al., 2015); NPHP is a relatively mild ciliopathy compared with skeletal ciliopathies. Three of the five families had missense variations in the CH domain, homozygous p.(I17S), homozygous p.(V125 M), and compound heterozygous p.(V125A) and p.(R155*). The other two variations were in the IFT54 middle region, a homozygous missense variation p.(M454R) and a homozygous frameshift variation p.(M459Mfs*3). Based on the observations in this study that disruption of a single interaction among multivalent interactions has a marginal effect on dynein 2 loading, point mutations in the CH domain or middle region are likely to have very limited effects on ciliary function. Indeed, ciliogenesis of fibroblasts from a patient with the IFT54(V125M) variation was nearly normal (Bizet et al., 2015). However, fibroblasts from a patient with a nucleotide variation c.1575+6T>G and a deduced protein change p.(M459Mfs*3) also appeared normal in terms of ciliogenesis (Bizet et al., 2015); this seems incompatible with the fact that IFT54(1–532) was not incorporated into the IFT-B complex (Fig. 2, lane 4) and its expression in *IFT54*-KO cells could not restore ciliogenesis (Fig. 1F). However, as the c.1575+6T>G variation is located at the end of exon 13, the nucleotide variation could affect splicing of this exon. If so, the predicted protein product is IFT54(Δ419-459), which is expected to retain the abilities to be incorporated into the IFT-B complex and to rescue ciliogenesis in *IFT54*-KO cells in view of the results of this study (Figs 1 and 2). Further studies, such as minigene splicing analysis to examine the effects of the nucleotide variation found in the patient on splicing, will be needed to resolve this apparent contradiction.

## MATERIALS AND METHODS

### Plasmids, antibodies, reagents, and cell lines

IFT54 constructs used in this study are listed in Table S1. In the current and previous studies (Hiyamizu et al., 2023; Katoh et al., 2016), we used a cDNA encoding human IFT54/TRAF3IP1 isoform 2 (625-amino acids; NP_001132962) instead of isoform 1 (691-amino acids; NP_056465), because the IFT54 proteins from most other vertebrate species correspond to isoform 2. The other constructs were described previously (Hiyamizu et al., 2023). Antibodies used in this study are listed in Table S2. Glutathione *S*-transferase (GST)-tagged anti-mCherry Nb (LaM-2 version) prebound to glutathione–Sepharose 4B beads were prepared as described previously (Ishida et al., 2021; Katoh et al., 2015). SAG was purchased from Enzo Life Sciences. hTERT-RPE1 cells (American Type Culture Collection, CRL-4000) were grown in DMEM/Ham's F-12 medium (Nacalai Tesque) supplemented with 10% fetal bovine serum (FBS) and 0.348% sodium bicarbonate at 37°C in 5% CO_2_. HEK293T cells (RIKEN BioResource Research Center, RBC2202) were cultured in DMEM with high glucose (Nacalai Tesque) supplemented with 5% FBS.

### Establishment of IFT54-KO cells and establishment of IFT54-KO cells stably expressing mCherry-fused IFT54 constructs

*IFT54*-KO cells were established from hTERT-RPE1 cells by essentially the same strategy using the CRISPR/Cas9 system as we previously described for the establishment of *WDR60*-KO cells (Hamada et al., 2018; Katoh et al., 2017). The single guide RNA (sgRNA) sequence targeting the exon of the human *IFT54* gene encoding the C-terminal coiled-coil region (Table S3) was designed using CRISPOR (Haeussler et al., 2016). A double-stranded oligonucleotide for the target sequence was inserted into the all-in-one sgRNA expression vector peSpCAS9(1.1)-2×sgRNA (Addgene 80768). hTERT-RPE1 cells were seeded onto a 12-well plate to approximately 1.5×10^5^ cells, and the next day, they were transfected with 1 µg of the sgRNA vector and 0.25 µg of the donor knock-in vector, pDonor-tBFP-NLS-Neo(universal) (Addgene 80767), using X-tremeGENE9 Reagent (Roche Applied Science). After selection in the presence of G418 (600 µg/ml), the cells with blue nuclear fluorescence were isolated. Genomic DNA extracted from the isolated cells was subjected to PCR using PrimeSTAR GXL Premix Fast DNA polymerase (Takara Bio) using three sets of primers (Table S2) to distinguish the following three states of integration of the donor vector: forward integration, reverse integration, and no integration with a small insertion or deletion (Fig. S1A). Direct sequencing of the PCR products was performed to confirm the disruption of both alleles of the *IFT54* gene; a small deletion resulting in a frameshift in one allele and an integration of the donor vector in the other allele (Fig. S1B). The resultant *IFT54*-KO clone (#IFT54-4-3) was used in this study.

Lentiviral vectors for mCherry-fused IFT54 constructs were prepared as described previously (Qiu et al., 2022; Takahashi et al., 2012). HEK293T cells were transfected with pRRLsinPPT-mCherry-IFT54 or its deletion construct along with the packaging vectors pRSV-REV, pMD2.g, and pMDLg/pRRE; kind gifts from Peter McPherson, McGill University (Thomas et al., 2009). The culture medium was replaced 8 h after transfection. Culture media containing lentiviral particles were collected at 24, 36, and 48 h after transfection, passed through a 0.45-µm filter, and centrifuged at 32,000×***g*** at 4°C for 4 h. Viral particles in the precipitates were resuspended in Opti-MEM (Thermo Fisher Scientific) and stored at −80°C until use. *IFT54*-KO cells expressing the mCherry-fused IFT54 construct were prepared by addition of the lentiviral suspension to the culture medium and processed for immunofluorescence analysis.

### Immunofluorescence analysis

To induce ciliogenesis, hTERT-RPE1 cells were grown to 100% confluence on coverslips, and serum-starved for 24 h in DMEM/F-12 containing 0.2% bovine serum albumin.

Immunofluorescence analysis was performed as described previously (Hiyamizu et al., 2023; Qiu et al., 2022). Cells were fixed and permeabilized with 3% paraformaldehyde at 37°C for 5 min, followed by 100% methanol for 5 min at −20°C, and then washed three times with phosphate-buffered saline. The fixed and permeabilized cells were blocked with 10% FBS, stained with antibodies diluted in 5% FBS (for experiments shown in Figs 1C–K, 3A–F, 4A–F, 5A–L), or stained with antibodies diluted in Can Get Signal Immunostain Solution A (Toyobo) (for experiments shown in Fig. 5N–Y). The immunostained cells were observed using an Axio Observer microscope (Carl Zeiss). Quantification analysis was performed as described previously (Qiu et al., 2022). Briefly, all images acquired under the same setting and saved in CZI file format were processed and analyzed using the ZEN3.1 microscope software (Carl Zeiss). A region of interest (ROI) was created by drawing a line along the signal of ARL13B within cilia using the Draw Spline Contour tool in the ZEN 3.1 imaging software, and the fluorescence intensity in the ROI was quantified. To measure fluorescence intensity at the tip and base of cilia, ROIs were created by drawing a circle at both the tip and the base of cilia using the Draw Circle tool in the ZEN 3.1 imaging software. To correct for local background intensity, the ROIs were duplicated and set to a nearby region. Statistical analyses were performed using GraphPad Prism 8 (Version 8.4.3; GraphPad Software, Inc.). To measure IFT88 staining intensities along individual cilia, ROIs were created by drawing a curve along cilia from the base (position of FOP staining) to the tip using a Draw Curve tool in the ZEN 3.1 imaging software, and the fluorescence intensity in the ROI was quantified. To correct for local background intensity, the ROI was duplicated and set in a nearby region.

### Coimmunoprecipitation analysis

*IFT54*-KO cells stably expressing an mCherry-fused IFT54 construct were grown to almost confluency on a 15-cm plate. The cells were lysed in 1 mL of lysis buffer (20 mM HEPES-KOH [pH 7.4], 3 mM MgCl_2_, 1 mM dithiothreitol, 100 mM KCl, 10% glycerol, 5 mM NaCl, and 0.1% Triton X-100) containing EDTA-free protease inhibitor cocktail (Nacalai Tesque) by placing on ice for 40 min. After centrifugation of the lysates at 16,000×***g*** at 4°C for 20 min, the supernatants were incubated with 40 µl of GST-tagged anti-mCherry Nb prebound to glutathione-Sepharose 4B beads. The beads were washed five times with the lysis buffer and boiled in SDS-PAGE sample buffer. The proteins bound to the beads were then separated by SDS-PAGE, and electroblotted onto an Immobilon-P membrane (Merck Millipore). The membrane was blocked in 5% skimmed milk and incubated sequentially with the primary antibody and the peroxidase-conjugated secondary antibody. Protein bands were detected with a Chemi-Lumi one L kit, Chemi-Lumi super kit or Chemi-Lumi ultra kit (Nacalai Tesque).

## Supplementary Material

10.1242/biolopen.059976_sup1 Supplementary information

## References

[BIO059976C1] Anvarian, Z., Mykytyn, K., Mukhopadhyay, S., Pedersen, L. B. and Christensen, S. T. (2019). Cellular signaling by primary cilia in development, organ function and disease. *Nat. Rev. Nephrol.* 15, 199-219. 10.1038/s41581-019-0116-9 30733609 PMC6426138

[BIO059976C2] Beales, P. L., Bland, E., Tobin, J. L., Bacchelli, C., Tuysuz, B., Hill, J., Rix, S., Pearson, C. G., Kai, M., Hartley, J. et al. (2007). *IFT80*, which encodes a conserved intraflagellar transport protein, is mutated in Jeune asphyxiating thoracic dystrophy. *Nat. Genet.* 39, 727-729. 10.1038/ng2038 17468754

[BIO059976C3] Bizet, A. A., Becker-Heck, A., Ryan, R., Weber, K., Filhol, E., Krug, P., Halbritter, J., Delous, M., Lasbennes, M. C., Linghu, B. et al. (2015). Mutations in TRAF3IP1/IFT54 reveal a new role for IFT proteins in microtubule stabilization. *Nat. Commun.* 6, 8666. 10.1038/ncomms9666 26487268 PMC4617596

[BIO059976C4] Boldt, K., van Reeuwijk, J., Lu, Q., Koutroumpas, K., Nguyen, T. M., Texier, Y., van Beersum, S. E. C., Horn, N., Willer, J. R., Mans, D. et al. (2016). An organelle-specific protein landscape identifies novel diseases and molecular mechanisms. *Nat. Commun.* 7, 11491. 10.1038/ncomms11491 27173435 PMC4869170

[BIO059976C5] Braun, D. A. and Hildebrandt, F. (2017). Ciliopathies. *Cold Spring Harb. Perspect. Biol.* 9, a028191. 10.1101/cshperspect.a028191 27793968 PMC5334254

[BIO059976C6] Funabashi, T., Katoh, Y., Okazaki, M., Sugawa, M. and Nakayama, K. (2018). Interaction of heterotrimeric kinesin-II with IFT-B-connecting tetramer is crucial for ciliogenesis. *J. Cell Biol.* 217, 2867-2876. 10.1083/jcb.201801039 29903877 PMC6080941

[BIO059976C7] Garcia-Gonzalo, F. R. and Reiter, J. F. (2017). Open sesame: how transition fibers and the transition zone control ciliary composition. *Cold Spring Harb. Perspect. Biol.* 9, a028134. 10.1101/cshperspect.a028134 27770015 PMC5287074

[BIO059976C8] Haeussler, M., Schönig, K., Eckert, H., Eschstruth, A., Mianné, J., Renaud, J. B., Schneider-Maunoury, S., Shkumatava, A., Teboul, L., Kent, J. et al. (2016). Evaluation of off-target and on-target scoring algorithms and integration into the guide RNA selection tool CRISPOR. *Genome Biol.* 17, 148. 10.1186/s13059-016-1012-2 27380939 PMC4934014

[BIO059976C9] Halbritter, J., Bizet, A. A., Schmidts, M., Porath, J. D., Braun, D. A., Gee, H. Y., McInerney-Leo, A. M., Krug, P., Filhol, E., Davis, E. E. et al. (2013). Defects in the IFT-B component IFT172 cause Jeune and Mainzer-Saldino syndromes in humans. *Am. J. Hum. Genet.* 93, 915-925. 10.1016/j.ajhg.2013.09.012 24140113 PMC3824130

[BIO059976C10] Hamada, Y., Tsurumi, Y., Nozaki, S., Katoh, Y. and Nakayama, K. (2018). Interaction of WDR60 intermediate chain with TCTEX1D2 light chain of the dynein-2 complex is crucial for ciliary protein trafficking. *Mol. Biol. Cell* 29, 1628-1639. 10.1091/mbc.E18-03-0173 29742051 PMC6080652

[BIO059976C11] Hirano, T., Katoh, Y. and Nakayama, K. (2017). Intraflagellar transport-A complex mediates ciliary entry and retrograde trafficking of ciliary G protein-coupled receptors. *Mol. Biol. Cell* 28, 429-439. 10.1091/mbc.e16-11-0813 27932497 PMC5341726

[BIO059976C12] Hirano, M., Ando, R., Shimozono, S., Sugiyama, M., Takeda, N., Kurokawa, H., Deguchi, R., Endo, K., Haga, K., Takai-Todaka, R. et al. (2022). A highly photostable and bright green fluorescent protein. *Nat. Biotechnol.* 40, 1132-1142. 10.1038/s41587-022-01278-2 35468954 PMC9287174

[BIO059976C13] Hiyamizu, S., Qiu, H., Vuolo, L., Stevenson, N., Shak, C., Heesom, K. J., Hamada, Y., Tsurumi, Y., Chiba, S., Katoh, Y. et al. (2023). Multiple interactions of the dynein-2 complex with the IFT-B complex are required for effective intraflagellar transport. *J. Cell Sci.* 136, jcs260462. 10.1242/jcs.260462 36632779 PMC10110421

[BIO059976C14] Ishida, Y., Kobayashi, T., Chiba, S., Katoh, Y. and Nakayama, K. (2021). Molecular basis of ciliary defects caused by compound heterozygous *IFT144/WDR19* mutations found in cranioectodermal dysplasia. *Hum. Mol. Genet.* 30, 213-225. 10.1093/hmg/ddab034 33517396

[BIO059976C15] Ishida, Y., Tasaki, K., Katoh, Y. and Nakayama, K. (2022). Molecular basis underlying the ciliary defects caused by IFT52 variations found in skeletal ciliopathies. *Mol. Biol. Cell* 33, ar83. 10.1091/mbc.E22-05-0188 35704471 PMC9582644

[BIO059976C16] Jordan, M. A. and Pigino, G. (2021). The structural basis of intraflagellar transport at a glance. *J. Cell Sci.* 134, jcs247163. 10.1242/jcs.247163 34137439

[BIO059976C17] Jordan, M. A., Diener, D. R., Stepanek, L. and Pigino, G. (2018). The cryo-EM structure of intraflagellar transport trains reveals how dynein is inactivated to ensure unidirectional anterograde movement in cilia. *Nat. Cell Biol.* 20, 1250-1255. 10.1038/s41556-018-0213-1 30323187

[BIO059976C18] Katoh, Y., Nozaki, S., Hartanto, D., Miyano, R. and Nakayama, K. (2015). Architectures of multisubunit complexes revealed by a visible immunoprecipitation assay using fluorescent fusion proteins. *J. Cell Sci.* 128, 2351-2362. 10.1242/jcs.168740 25964651

[BIO059976C19] Katoh, Y., Terada, M., Nishijima, Y., Takei, R., Nozaki, S., Hamada, H. and Nakayama, K. (2016). Overall architecture of the intraflagellar transport (IFT)-B complex containing Cluap1/IFT38 as an essential component of the IFT-B peripheral subcomplex. *J. Biol. Chem.* 291, 10962-10975. 10.1074/jbc.M116.713883 26980730 PMC4900248

[BIO059976C20] Katoh, Y., Michisaka, S., Nozaki, S., Funabashi, T., Hirano, T., Takei, R. and Nakayama, K. (2017). Practical method for targeted disruption of cilia-related genes by using CRISPR/Cas9-mediated homology-independent knock-in system. *Mol. Biol. Cell* 28, 898-906. 10.1091/mbc.e17-01-0051 28179459 PMC5385939

[BIO059976C21] Kopinke, D., Norris, A. M. and Mukhopadhyay, S. (2021). Developmental and regenerative paradigms of cilia regulated hedgehog signaling. *Sem. Cell Dev. Biol.* 110, 89-103. 10.1016/j.semcdb.2020.05.029 PMC7736055 32540122

[BIO059976C22] Kozminski, K. G., Johnson, K. A., Forscher, P. and Rosenbaum, J. L. (1993). A motility in the eukaryotic flagellum unrelated to flagellar beating. *Proc. Natl. Acad. Sci. USA* 90, 5519-5523. 10.1073/pnas.90.12.5519 8516294 PMC46752

[BIO059976C23] Kozminski, K. G., Beech, P. L. and Rosenbaum, J. L. (1995). The *Chlamydomonas* kinesin-like protein FLA10 is involved in motility associated with the flagellar membrane. *J. Cell Biol.* 131, 1517-1527. 10.1083/jcb.131.6.1517 8522608 PMC2120669

[BIO059976C24] Lacey, S. E., Foster, H. E. and Pigino, G. (2023). The molecular structure of IFT-A and IFT-B in anterograde intraflagellar transport trains. *Nat. Struct. Mol. Biol.* 30, 584-593. 10.1038/s41594-022-00905-5 36593313 PMC10191852

[BIO059976C25] McInerney-Leo, A. M., Harris, J. E., Marshall, M. S., Gardiner, B., Kinning, E., Leong, H. Y., McKenzie, F., Ong, W. P., Vodopiutz, J., Wicking, C. et al. (2015). Whole exome sequencing is an efficient, sensitive and specific method for determining the genetic cause of short-rib thoracic dystrophies. *Clin. Genet.* 88, 550-557. 10.1111/cge.12550 25492405

[BIO059976C26] Nachury, M. V. and Mick, D. U. (2019). Establishing and regulating the composition of cilia for signal transduction. *Nat. Rev. Mol. Cell Biol.* 20, 389-405. 10.1038/s41580-019-0116-4 30948801 PMC6738346

[BIO059976C27] Nakayama, K. and Katoh, Y. (2020). Architecture of the IFT ciliary trafficking machinery and interplay between its components. *Crit. Rev. Biochem. Mol. Biol.* 55, 179-196. 10.1080/10409238.2020.1768206 32456460

[BIO059976C28] Omori, Y., Zhao, C., Saras, A., Mukhopadhyay, S., Kim, W., Furukawa, T., Sengupta, P., Veraksa, A. and Malicki, J. (2008). *elipsa* is an early determinant of ciliogenesis that links the IFT particle to membrane-associated small GTPase Rab8. *Nat. Cell Biol.* 10, 437-444. 10.1038/ncb1706 18364699

[BIO059976C29] Petriman, N. A., Loureiro-López, M., Taschner, M., Zacharia, N. K., Georgieva, M. M., Boegholm, N., Wang, J., Mourão, A., Russell, R. B., Andersen, J. S. et al. (2022). Biochemically validated structural model of the 15-subunit IFT-B complex. *EMBO J.* 41, e112440. 10.15252/embj.2022112440 36354106 PMC9753473

[BIO059976C30] Prevo, B., Scholey, J. M. and Peterman, E. J. G. (2017). Intraflagellar transport: mechanisms of motor action, cooperation, and cargo delivery. *FEBS J.* 284, 2905-2931. 10.1111/febs.14068 28342295 PMC5603355

[BIO059976C31] Qiu, H., Tsurumi, Y., Katoh, Y. and Nakayama, K. (2022). Combinations of deletion and missense variations of the dynein-2 DYNC2LI1 subunit found in skeletal ciliopathies cause ciliary defects. *Sci. Rep.* 12, 31. 10.1038/s41598-021-03950-0 34997029 PMC8742128

[BIO059976C32] Reiter, J. F. and Leroux, M. R. (2017). Genes and molecular pathways underpinning ciliopathies. *Nat. Rev. Mol. Cell Biol.* 18, 533-547. 10.1038/nrm.2017.60 28698599 PMC5851292

[BIO059976C33] Rosenbaum, J. L. and Witman, G. B. (2002). Intraflagellar transport. *Nat. Rev. Mol. Cell Biol.* 3, 813-825. 10.1038/nrm952 12415299

[BIO059976C34] Schmidts, M. (2014). Clinical genetics and pathobiology of ciliary chondrodysplasias. *J. Pediatr. Genet.* 3, 49-64. 10.3233/PGE-14089 PMC4262788 25506500

[BIO059976C35] Shak, C., Vuolo, L., Uddin, B., Katoh, Y., Brown, T., Mukhopadhyay, A. G., Heesom, K., Roberts, A. J., Stevenson, N., Nakayama, K. et al. (2023). Disease-associated mutations in WDR34 lead to diverse impacts on the assembly and function of dynein-2. *J. Cell Sci.* 136, jcs260073. 10.1242/jcs.260073 36268591 PMC9687537

[BIO059976C36] Takahara, M., Katoh, Y., Nakamura, K., Hirano, T., Sugawa, M., Tsurumi, Y. and Nakayama, K. (2018). Ciliopathy-associated mutations of IFT122 impair ciliary protein trafficking but not ciliogenesis. *Hum. Mol. Genet.* 27, 516-528. 10.1093/hmg/ddx421 29220510

[BIO059976C37] Takahashi, S., Kubo, K., Waguri, S., Yabashi, A., Shin, H.-W., Katoh, Y. and Nakayama, K. (2012). Rab11 regulates exocytosis of recycling vesicles at the plasma membrane. *J. Cell Sci.* 125, 4049-4057. 22685325 10.1242/jcs.102913

[BIO059976C38] Taschner, M. and Lorentzen, E. (2016). The intraflagellar transport machinery. *Cold Spring Harb. Perspect. Biol.* 8, a028092. 10.1101/cshperspect.a028092 27352625 PMC5046692

[BIO059976C39] Taschner, M., Weber, K., Mourão, A., Vetter, M., Awasthi, M., Stiegler, M., Bhogaraju, S. and Lorentzen, E. (2016). Intraflagellar transport proteins 172, 80, 57, 54, 38, and 20 form a stable tubulin-binding IFT-B2 complex. *EMBO J.* 35, 773-790. 10.15252/embj.201593164 26912722 PMC4818760

[BIO059976C40] Taylor, S. P., Dantas, T. J., Duran, I., Wu, S., Lachman, R. S., University of Washington Center for Mendelian Genomics Consortium, Nelson, S. F., Cohn, D. H., Vallee, R. B. and Krakow, D. (2015). Mutations in *DYNC2LI1* disrupt cilia function and cause short rib polydactyly syndrome. *Nat. Commun.* 6, 7092. 10.1038/ncomms8092 26077881 PMC4470332

[BIO059976C41] Thomas, S., Ritter, B., Verbich, D., Sanson, C., Bourbonnière, L., McKinney, R. A. and McPherson, P. S. (2009). Intersectin regulates dendritic spine development and somatodendritic endocytosis but not synaptic vesicle recycling in hippocampal neurons. *J. Biol. Chem.* 284, 12410-12419. 10.1074/jbc.M809746200 19258322 PMC2673308

[BIO059976C42] Toropova, K., Mladenov, K. and Roberts, A. J. (2017). Intraflagellar transport dynein is autoinhibited by trapping of its mechanical and track-binding elements. *Nat. Struct. Mol. Biol.* 24, 461-468. 10.1038/nsmb.3391 28394326 PMC5420313

[BIO059976C43] Toropova, K., Zalyte, R., Mukhopadhyay, A. G., Mladenov, M., Carter, A. P. and Roberts, A. J. (2019). Structure of the dynein-2 complex and its assembly with intraflagellar transport trains. *Nat. Struct. Mol. Biol.* 26, 823-829. 10.1038/s41594-019-0286-y 31451806 PMC6774794

[BIO059976C44] Tsurumi, Y., Hamada, Y., Katoh, Y. and Nakayama, K. (2019). Interactions of the dynein-2 intermediate chain WDR34 with the light chains are required for ciliary retrograde protein trafficking. *Mol. Biol. Cell* 30, 658-670. 10.1091/mbc.E18-10-0678 30649997 PMC6589695

[BIO059976C45] van den Hoek, H., Klena, N., Jordan, M. A., Viar, G. A., Righetto, R. D., Schaffer, M., Erdmann, P. S., Wan, W., Geimer, S., Plitzko, J. M. et al. (2022). In situ architecture of the ciliary base reveals the stepwise assembly of intraflagellar transport trains. *Science* 377, 543-548. 10.1126/science.abm6704 35901159

[BIO059976C46] Vuolo, L., Stevenson, N. L., Heesom, K. J. and Stephens, D. J. (2018). Dynein-2 intermediate chains play crucial but distinct roles in primary cilia formation and function. *eLife* 7, e39655. 10.7554/eLife.39655 30320547 PMC6211827

[BIO059976C47] Vuolo, L., Stevenson, N. L., Mukhopadhyay, A. G., Roberts, A. J. and Stephens, D. J. (2020). Cytoplasmic dynein-2 at a glance. *J. Cell Sci.* 133, jcs240614. 10.1242/jcs.240614 32229580

[BIO059976C48] Webb, S., Mukhopadhyay, A. G. and Roberts, A. J. (2020). Intraflagellar transport trains and motors: insights from structure. *Sem*. *Cell Dev. Biol.* 107, 82-90. 10.1016/j.semcdb.2020.05.021 PMC7561706 32684327

[BIO059976C49] Wu, C., Li, J., Peterson, A., Tao, K. and Wang, B. (2017). Loss of dynein-2 intermediate chain Wdr34 results in defects in retrograde ciliary protein trafficking and Hedgehog signaling in the mouse. *Hum. Mol. Genet.* 26, 2386-2397. 10.1093/hmg/ddx127 28379358 PMC6075199

[BIO059976C50] Zhang, W., Taylor, S. P., Ennis, H. A., Forlenza, K. N., Duran, I., Li, B., Ortiz Sanchez, J. A., Nevarez, L., Nickerson, D. A., Bamshad, M. et al. (2018). Expanding the genetic architecture and phenotypic spectrum in the skeletal ciliopathy. *Hum. Mut.* 39, 152-166. 10.1002/humu.23362 29068549 PMC6198324

[BIO059976C51] Zhu, X., Liang, Y., Gao, F. and Pan, J. (2017). IFT54 regulates IFT20 stability but is not essential for tubulin transport during ciliogenesis. *Cell. Mol. Life Sci.* 74, 3425-3437. 10.1007/s00018-017-2525-x 28417161 PMC11107664

[BIO059976C52] Zhu, X., Wang, J., Li, S., Lechtreck, K. and Pan, J. (2021). IFT54 directly interacts with kinesin-II and IFT dynein to regulate anterograde intraflagellar transport. *EMBO J.* 40, e105781. 10.15252/embj.2020105781 33368450 PMC7917553

