# Supplementary material for: Dynein-2–driven intraciliary retrograde trafficking indirectly requires multiple interactions of IFT54 in the IFT-B complex with the dynein-2 complex
Source: Biol Open. 2023 Jun 28;12(7):bio059976. doi: 10.1242/bio.059976 (PMC10320715; doi:10.1242/bio.059976)
Supplement: Supplementary information [file biolopen-12-059976-s1.pdf]

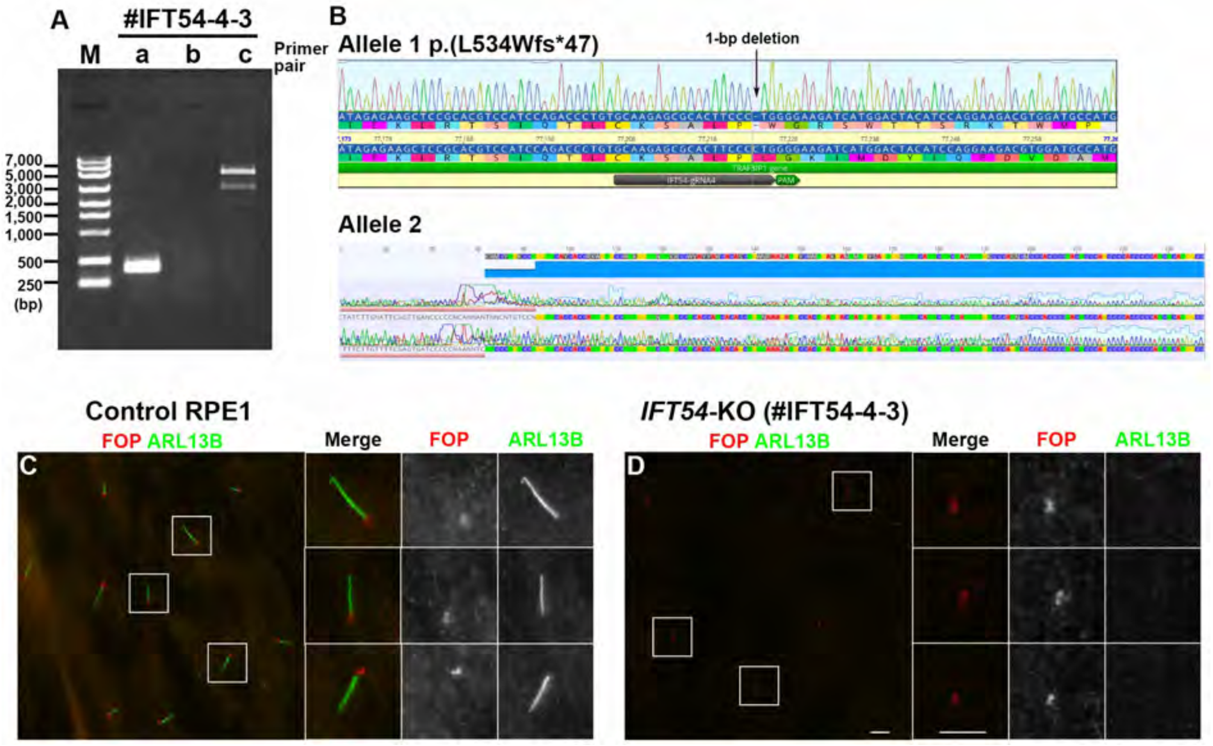

Fig. S1. Analyses of *IFT54* genomic sequences of the *IFT54*-KO cell line

(A) Genomic DNA extracted from the *IFT54*-KO cell line #IFT54-4-3 was subjected to PCR using primer sets (a, b, and c; Table S3) to detect alleles with forward (b) or reverse (c) integration of the donor knock-in vector or no insertion/small indel (a). (B) The amplified DNAs with no insertion/small indel (allele 1) and reverse integration of the donor vector (allele 2) were subjected to sequence analysis. Note that for allele 2, as an unrelated long sequence from human chromosome 1 (LOC126805873), in addition to the knock-in vector sequence, was inserted, we could not determine the exact insertion site of the knock-in vector. However, as the abnormal cilia-lacking phenotype of the cell line #IFT54-4-3 was rescued by exogenous expression of IFT54(WT) (see Fig. 1D, E), the abnormal phenotype is not likely to result from an off-target effect. (C, D) Control RPE1 cells (C) and the #IFT54-4-3 cell line (D) were cultured under serum-starved conditions for 24 h to induce ciliogenesis, and doubly immunostained for FOP and ARL13B. Enlarged (2.5-fold) images of the boxed regions are shown on the right. Scale bars, 5 µm. The #IFT54-4-3 cell line could not form cilia, consistent with the disruption of both *IFT54* alleles.

Biology Open • Supplementary information

Fig. S2: Uncropped images

Fig. 2: mChe-IFT54

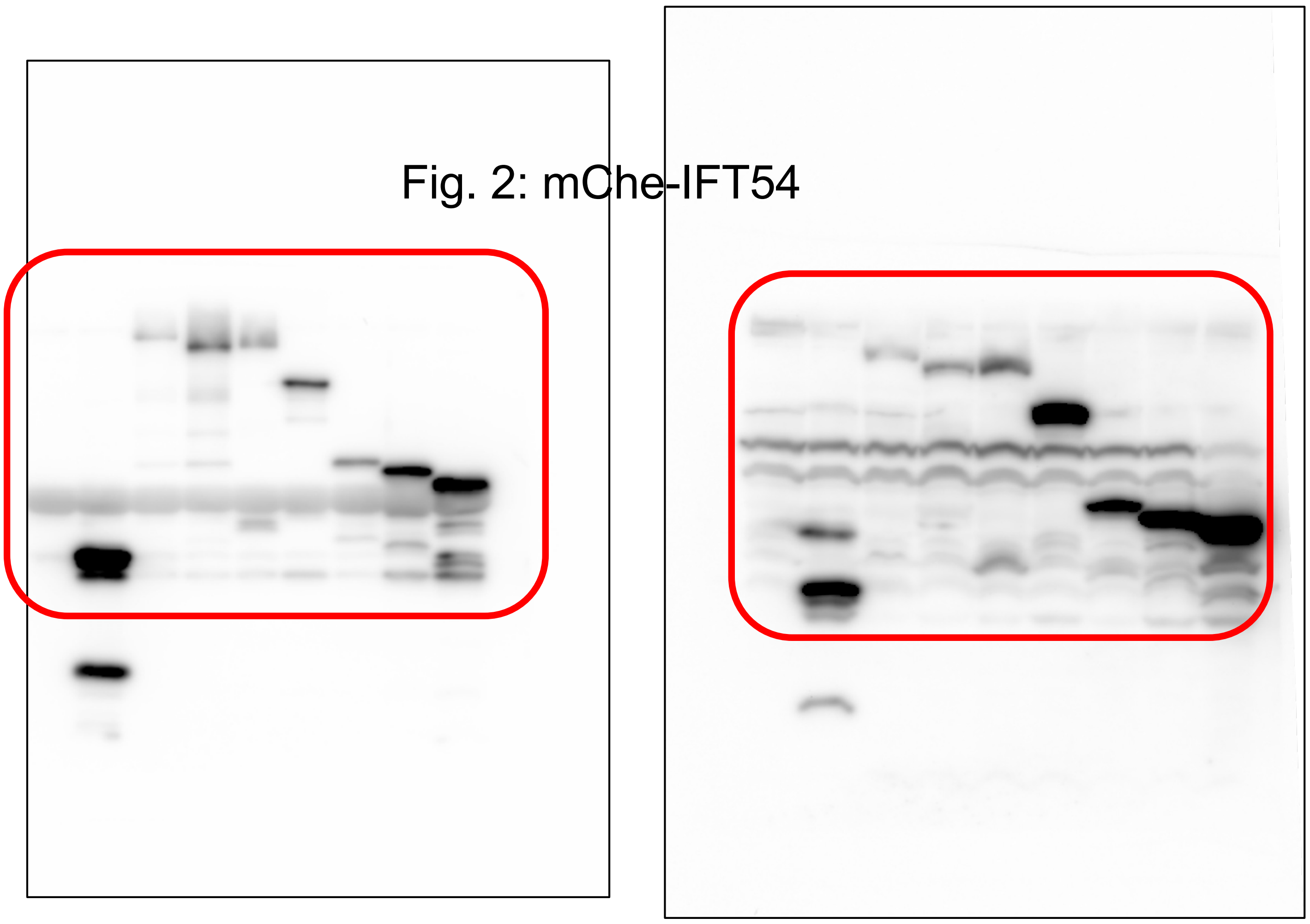

Fig. 2: IFT88

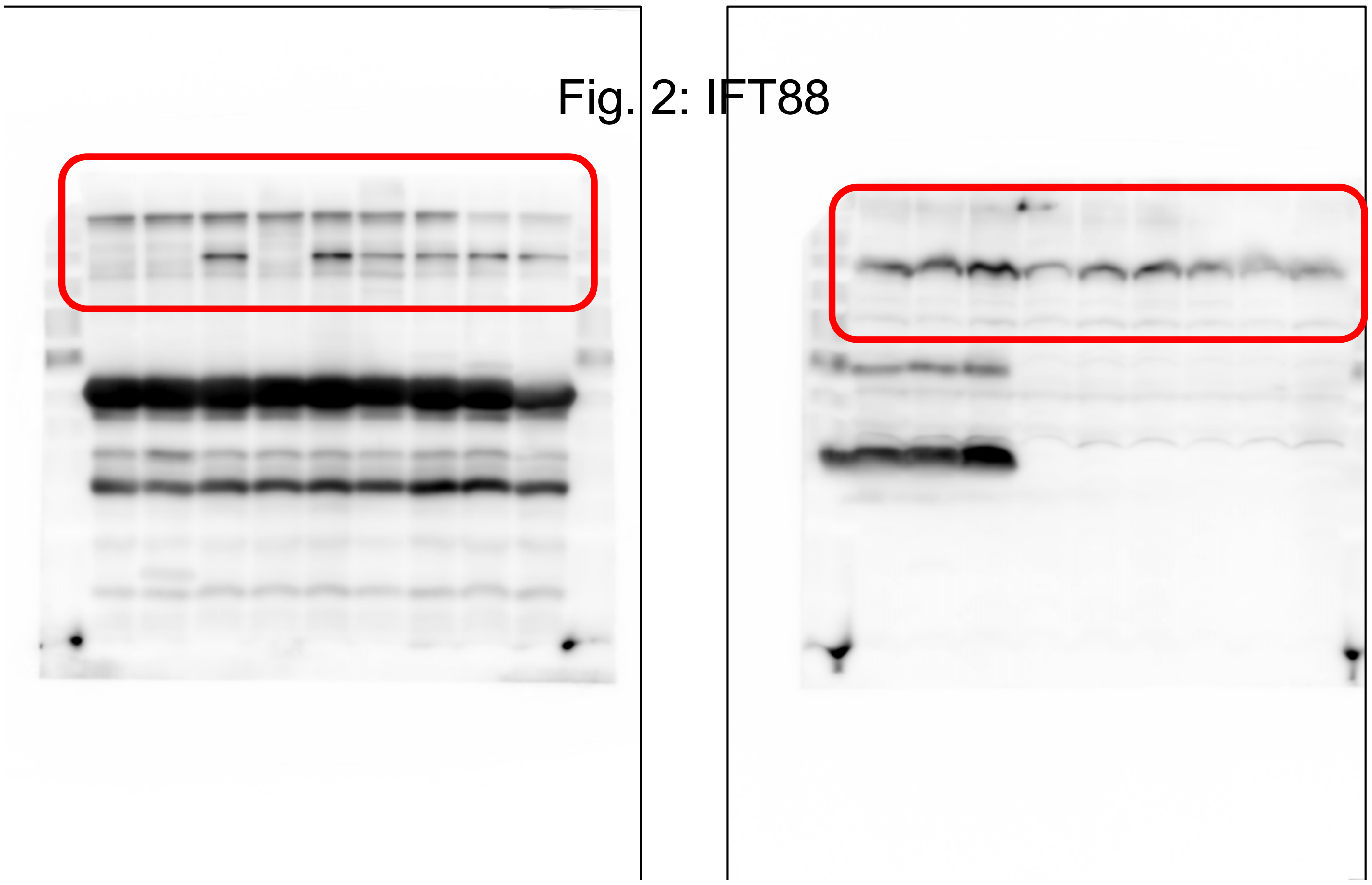

Biology Open • Supplementary information

Fig. S2: Uncropped images

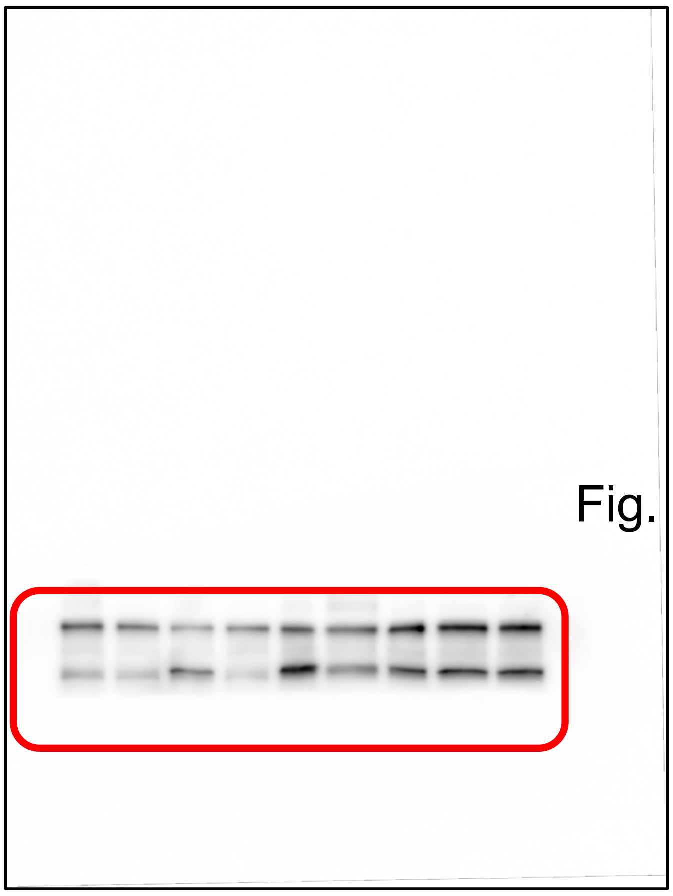

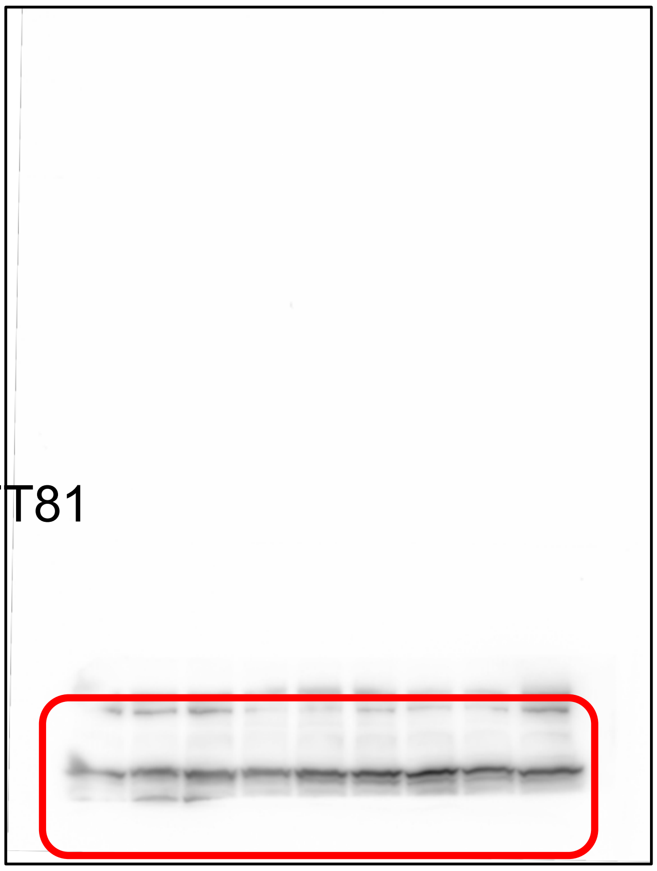

Fig. 2: IFT81

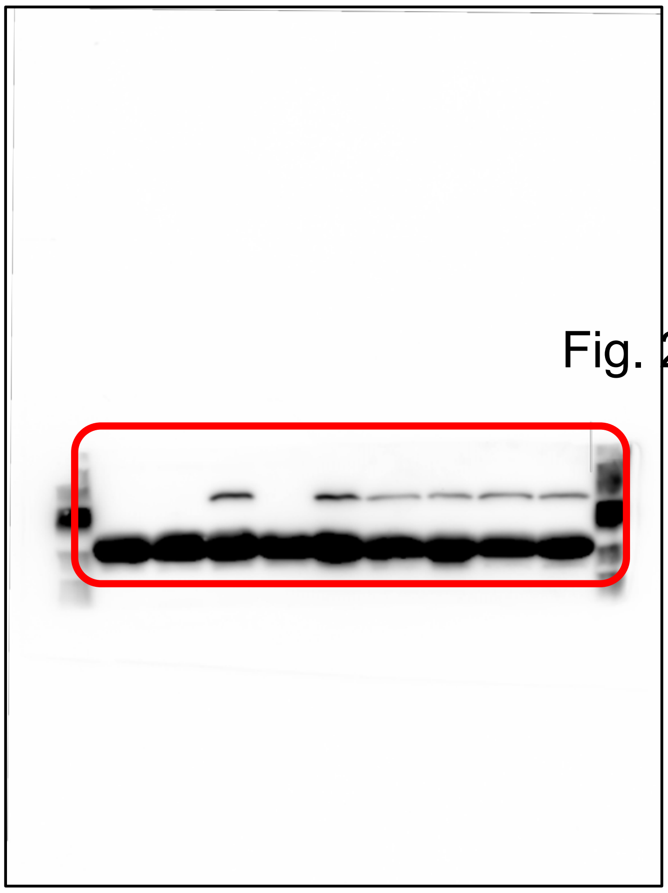

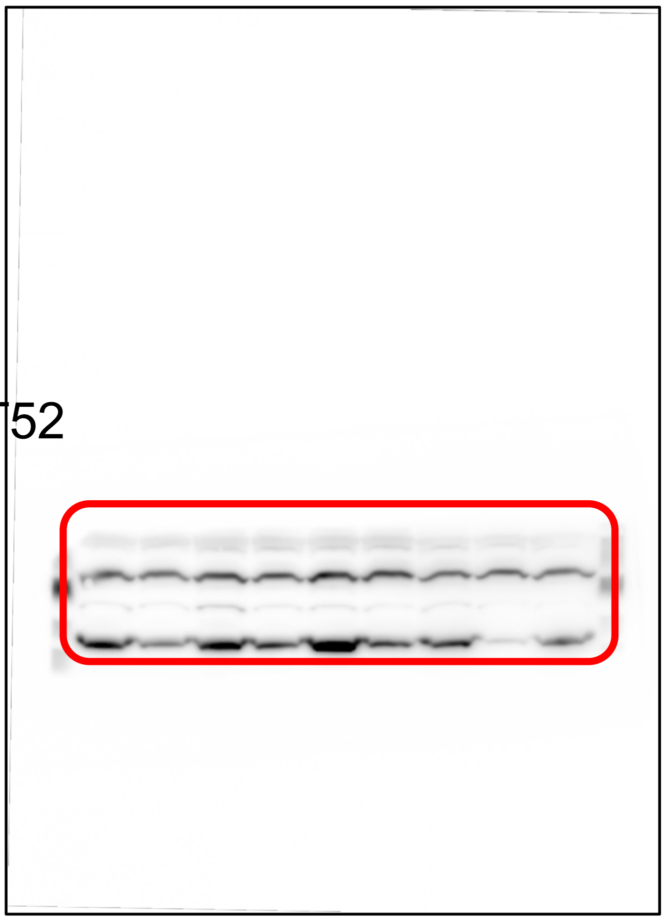

Fig. 2: IFT52

Biology Open • Supplementary information

Fig. S2: Uncropped images

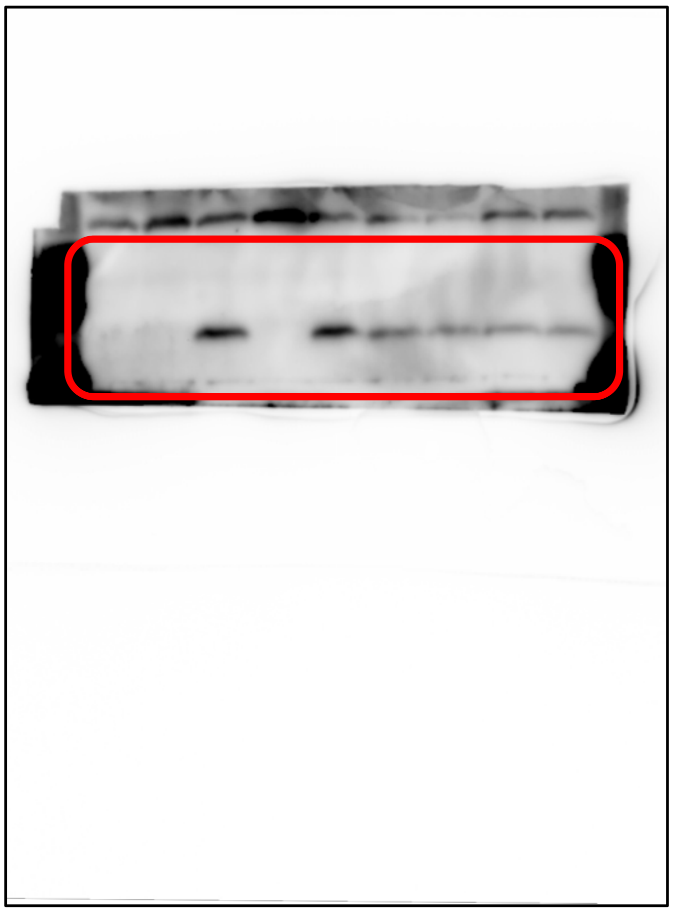

Fig. 2: IFT25

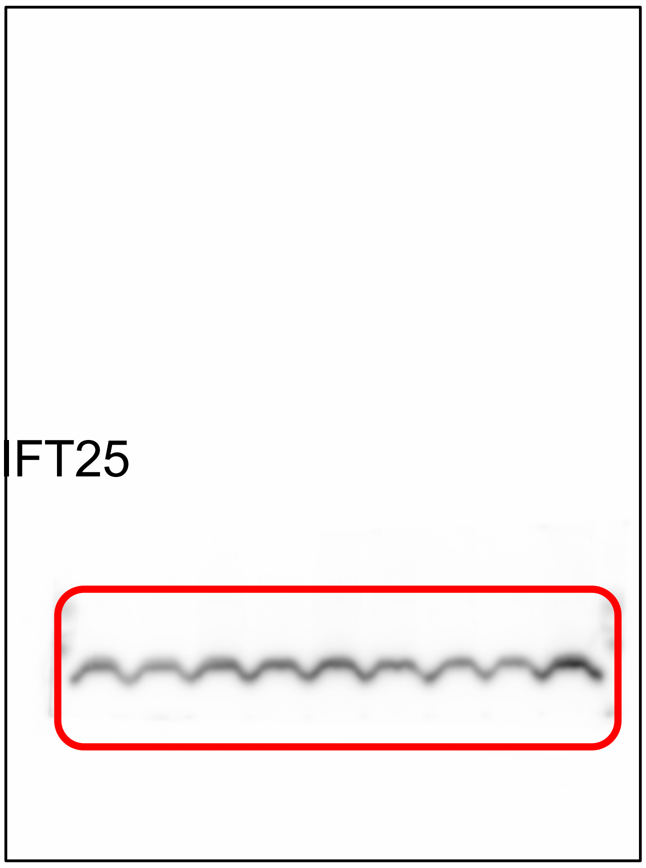

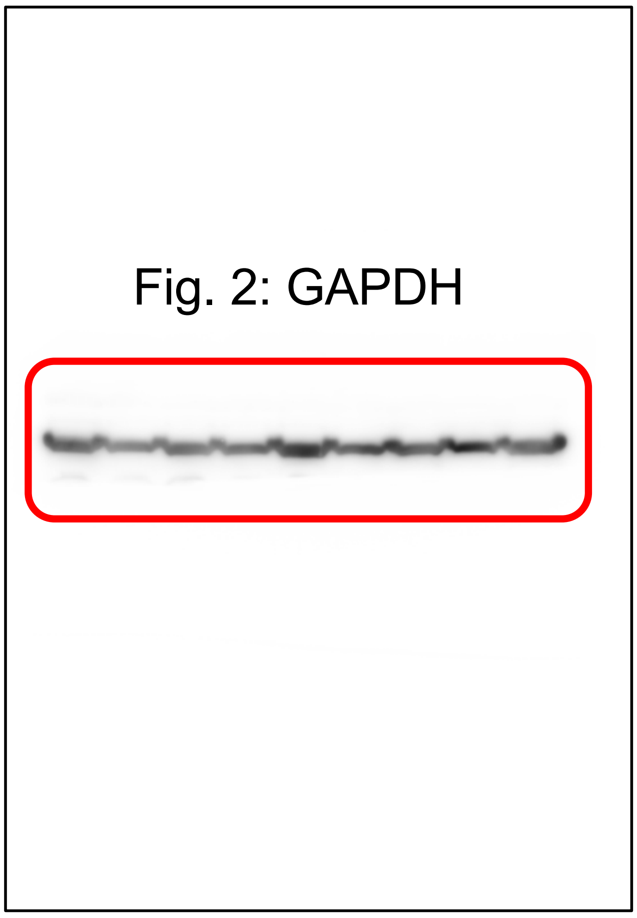

Fig. S1A

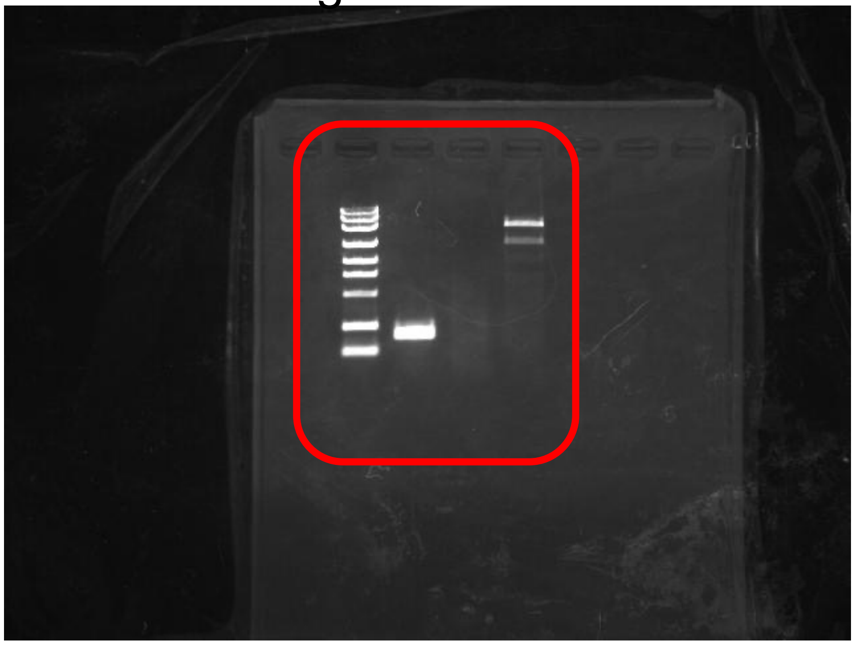

Biology Open • Supplementary information

Table S1. Plasmids used in this study

Vector	Insert *	Reference
pRRLsinPPT-mCherry-C-IRES-Blast	IFT54	This study
pRRLsinPPT-mCherry-C-IRES-Blast	IFT54(1–532)	This study
pRRLsinPPT-mCherry-C-IRES-Blast	IFT54(135–625)	This study
pRRLsinPPT-mCherry-C-IRES-Blast	IFT54(335–625)	This study
pRRLsinPPT-mCherry-C-IRES-Blast	IFT54(488–625)	This study
pRRLsinPPT-mCherry-C-IRES-Blast	IFT54(507–625)	This study
pRRLsinPPT-mCherry-C-IRES-Blast	IFT54(533–625)	This study
pGEX-6P1	Anti-mCherry-Nanobody (LaM-2)	Ishida et al., 2021

* All cDNA inserts except for that of anti-mCherry Nb are of human origin.

Biology Open • Supplementary information

Table S2. Antibodies used in this study

Antibody	Manufacturer	Clone/catalog number or reference number	Dilution (purpose)
Polyclonal rabbit anti-IFT25	Proteintech	15732-1-AP	1:1,000 (IB)
Polyclonal rabbit anti-IFT52	Proteintech	17534-1-AP	1:1,000 (IB)
Polyclonal rabbit anti-IFT81	Proteintech	11744-1-AP	1:1,000 (IB)
Polyclonal rabbit anti-IFT88	Proteintech	13967-1-AP	1:500 (IF), 1:1,000 (IB)
Polyclonal rabbit anti-IFT140	Proteintech	17460-1-AP	1:500 (IF)
Polyclonal rabbit anti-GPR161	Proteintech	13398-1-AP	1:200 (IF)
Polyclonal rabbit anti-ARL13B	Proteintech	17711-1-AP	1:500 (IF)
Monoclonal mouse anti-ARL13B	Abcam	N295B/66	1:500 (IF)
Monoclonal mouse anti-FOP	Abnova	2B1	1:10,000 (IF)
Monoclonal mouse anti-Smoothened	Santa Cruz	sc-166685	1:100 (IF)
Monoclonal mouse anti-RFP	MBL	3G5	1:1,000 (IF)
Monoclonal mouse anti-GAPDH	Ambion	6C5	1:10,000 (IB)
Polyclonal rabbit anti-mCherry	Proteintech	26765-1-AP	1:10,000 (IB)
AlexaFluor-conjugated secondary	Molecular Probes	A11034, A21127, A21131, A21147, A21241, A21242, A21245	1:1,000 (IF)
Peroxidase-conjugated secondary	Jackson ImmunoResearch	115-035-166, 111-035-144	1:3,000 (IB)

IF, immunofluorescence; IB, immunoblotting

Table S3. Oligodeoxyribonucleotides used in this study

Name	Sequence
IFT54-gRNA#4-S	5'-CACCGCAAGAGCGCACTTCCCCTG-3'
IFT54-gRNA#4-AS	5'-AAACCAGGGGAAGTGCGCTCTTGC-3'
IFT54-Genome-#4-FW	5'-GCAGTGCTGTGTCCTCTGAT-3'
IFT54-Genome-#4-RV	5'-TGCCACATCTGCAGCTCATT-3'
pTagBFP-N-RV2	5'-CGTAGAGGAAGCTAGTAGCCAGG-3'

S, sense; AS, antisense; FW, forward; RV, reverse
